# Establishment of the model system between phytochemicals and gene expression profiles in Macrosclereid cells of *Medicago truncatula*

**DOI:** 10.1038/s41598-017-02827-5

**Published:** 2017-05-31

**Authors:** Fuyou Fu, Wentao Zhang, Yuan-Yuan Li, Hong Li Wang

**Affiliations:** 1 Biology Department University of Arkansas at Little Rock, 2801 South University Ave. Little Rock, Arkansas, 72204 USA; 2 Aquatic and Crop Resources Development, National Research Council of Canada, 110 Gymnasium Place, Saskatoon, SK S7N 0W9 Canada; 3 Nutrition Research Institute, University of North Carolina at Chapel Hill, 500 Laureate Way, Kanaplolis, 28081 NC USA

## Abstract

Macrosclereid cells, which are a layer in the seed coat of *Medicago truncatula*, accumulate large amounts of phytochemicals during their development. But little is known about the complex and dynamic changes during macrosclereid cell development. To characterize the phytochemicals and the related gene expression during the development of *M*. *truncatula* macrosclereid cells, a high performance liquid chromatography-mass spectrometry (HPLC-MS) assay and microarray study were conducted on transcriptome changes from macrosclereid cell during seed development. A total of 16 flavonoids by HPLC-MS and 4861 genes exhibited significant differences at transcript levels by microarray analysis were identified for macrosclerid cells at six different time points during seed development. 815 abiotic and biotic stress genes, 223 transcriptional factors (TFs), and 155 annotated transporter proteins exhibited differential expression during the development of macrosclereid cells. A total of 102 genes were identified as involved in flavonoid biosynthesis, phenypropanoid biosynthesis, and flavone and flavonol biosynthesis. We performed a weighted gene co-regulatory network (WGCNA) to analyze the gene-flavonoid association and rebuilt the gene regulatory network during macrosclereid cell development. Our studies revealed that macrosclereid cells are, beside as the first barrier of defense against diseases, an excellent model system to investigate the regulatory network that governs flavonoid biosynthesis.

## Introduction

*Medicago truncatula*, a member of the *Fabaceae*, has garnered considerable attention as a genomic and molecular model system for legume biological studies^1^, due to its small genome size, phenotypic variations, small size, short life cycle, and completed genomic DNA sequence (www.medicago.org). To date, *M*. *truncatula* has been broadly used as a model plant for the study of root-rhizobial interactions, molecular regulation of nodule development^2, 3^, lignin biosynthesis^4, 5^, seed coat development^6^, and flavonoid biosynthesis and transport^7–9^. However, few studies have focused on the development of *M*. *truncatula* MC (MC), which are a specialized cell layer in the seed coat^10^.

In angiosperms, a mature seed consists of an embryo, a seed coat, and an endosperm. Many previous studies focused on embryo and endosperm development^11, 12^ and seed coat color control^8, 13, 14^. In fact, the seed coat plays crucial roles in all species, i.e. protecting the embryo, limiting desiccation during dormancy and germination, and promoting seed dispersal^15^. In *M*. *truncatula*, the seed coat of *M*. *truncatula* is composed of several layers of specialized cells, including a uniform palisade layer of MC called the Malpighian layer^10^. These MC are radially elongated, and are covered by a thick cuticle layer on their outer surface. In order to reveal the development of MC and their role in mature seeds, we studied the cell development by light microscope and identified the phytochemicals present in MC by HPLC-MS.

*Arabidopsis thaliana* is a widely used model system for genetic analysis, and also has been used to study seed coat development. Several key genes involved in seed coat differentiation and proanthocyanidin (PA) biosynthesis regulation have been identified and characterized through studies of *Arabidopsis* seed coat mutants, and the PA biosynthetic pathway has been largely elucidated^14, 16–18^. In *M*. *truncatula*, several genes involved in seed coat differentiation, such as *MATE1* ^8^ and *MATE2* ^9^, have been identified and characterized. Many genes involved in seed coat development, such as *TT1*, *TT3*, *TT4*, *TT5*, *TT6*, *TT10* ^19^, *TTG1* ^20^, *TT2* ^21^, *TTG2* ^22^, *TT8* ^23^, *TT7* ^24^, *TT12* ^8, 25^, and *TT19* ^26^, have been identified using forward-genetic mutant screens. Although much is known about seed coat development in *Arabidopsis*, *M*. *truncatula*, and other plants, many gaps remain in our understanding of this process. More over, it is extremely challenging to identify mutants with no visible phenotype, such as those with minor changes in metabolic processes. Global gene expression analysis is regarded as a useful alternative approach to identify additional genes and gene regulatory networks that are involved in seed coat development.

Although gene expression profiles have been applied to seed coat development studies for *Arabidopsis* ^27, 28^ and *M*. *truncatula* ^13, 29^, these studies mainly focused on providing some insights into seed coat development. A global analysis of gene expression during seed coat development, particularly during macrosclereid development, has not yet been reported in *M*. *truncatula*. In the present study, we monitored morphological changes in developing MC using light microscopy and tracked the accumulation of flavonoids using HPLC-UV-MS. Furthermore, we conducted a comprehensive global analysis of gene expression in developing *M*. *truncatula* MC. Our studies indicated that TFs and structural genes involved in regulation the accumulation of flavonoid compounds in MC. The results of this study provide some insight into the distinct chemistry of developing MC.

## Materials and Methods

### Reagents and standards

LC-MS reagents were obtained from Fisher Scientific (Rockford, IL, USA) and ultra-pure water was generated using the model Milli-Q Plus System (Billerica, MA, USA). Flavonoid standards were obtained from Indofine (Somerville, NJ, USA), Sigma-Aldrich (St. Louis, MO), and Chromax (Irvine, CA). All standards (Table S1) were previously described by Fu *et al*.^30^.

### Plant material

Plants (*Medicago truncatula*, ecotype A17) were grown in pots containing a mixture of sand and perlite (2:8) under controlled growth chamber conditions with an average temperature of 23 °C, 50% humidity, and a 16-h photoperiod. Plants were watered/fertilized using a solution containing one tablespoon of Scotts Miracle-Gro per gallon water (Scotts Miracle-Gro Company, Marysville, OH, USA).

### Pod harvest and MC isolation

Randomly selected flowers were tagged (using small different colored tags and strings) on the day after pollination^10^. Whole pods were collected at various days post pollination (DPP; from 6 to 27 DPP). The seeds were immediately extracted from the harvested pods on ice. MC were isolated from the seed using an Olympus-SZX16 (10×, Olympus America Inc., Melville, NY, USA) on ice and rapidly placed in liquid nitrogen and stored at −80 °C for LC-ESI-MS and gene expression analyses.

### Tissue preparation and light microscopy observations

Tissues were prepared and light microscopy observations were made as previously described^10^. All sections were stained with Toluidine Blue O at pH 4.4 and observed with an Olympus-BH2 microscope (Olympus America Inc., Melville, NY, USA). Digital photomicrographs were acquired using a SPOT Insight Camera (Diagnostic Instruments, Inc., Sterling Heights, MI, USA).

### Flavonoid extraction

Frozen MC (100 mg per aliquot in fresh weight) were homogenized in 80% 1 ml methanol, and the suspension was placed in an ultrasonic bath for 1 h. The extract was centrifuged at 13000 rpm for 20 min at room temperature and the supernatant was filtered using 4.6 µm pore-diameter filters (Fisher Scientific, St. Louis, USA). The filtered solution was subjected to LC-MS analysis. The insoluble PAs (in-PAs) measurement was followed the method as previously described by Liang *et al*.^31^.

### HPLC-ESI-MS instrumentation

Phytochemical constituents were analyzed using an LC-MS/MS system comprised by an Agilent 1100 HPLC system (Hewlett-Packard, Palo Alto, CA, USA) with DAD detector and a Bruker Esquire 3000 ion-trap mass spectrometer (Bruker Daltonics, Bremen, Germany). The separation of analytes was carried out on a C18 column (4.6 × 250 mm, 5 µm) from Grace (Grace, Maryland, USA). The detection wavelength for UV signal was acquired at 600 nM, and the scan range was from 200 to 600 nm. The mobile phase of HPLC consisted of (A) water containing 0.1% formic acid (v/v), and (B) acetontrile, and the elution gradient was set as: 0 to 5 min, isocratic 95% A and 5% B; 5 to 10 min, isocratic 10% B; 10 to 17 min, isocratic 17% B; 17 to 25 min, isocratic 25% B; 25 to 30 min, isocratic 30% B; 30 to 55 min, isocratic 55% B; 55 to 65 min, isocratic 70% B; 65 to 70 min, isocratic 5% B; and 70 to 75 min, isocratic 95% A and 5% B. The flow rate was at 0.8 ml min^−1^ and the column temperature was maintained at 25 °C. LC-MS data was acquired in negative mode with mass range from 50 to 2200 m/z. The key parameters for ion-trap mass spectrometer (ITMS) were set as follows: ion source voltage, 3.5 kV; capillary temperature, 350 °C; and ion current control (ICC), 10,000 with a maximum acquisition time of 100 ms. The MS/MS data was obtained in a manual mode for targeted mass using an isolation width of 2.0, a fragmentation amplitude of 2.2, and a threshold set at 6000.

### RNA isolation and qRT-PCR

RNA was extracted from frozen MC using the Spectrum™ Plant Total RNA Kit with on-column DNaseI digestion (Sigma-Aldrich, St. Louis, USA). For cDNA synthesis, 1 µg of total RNA was reverse transcribed using the iScript cDNA Synthesis Kit (Bio-Rad Laboratories, USA), which contains MMLV-derived reverse transcriptase and is primed with random primers. Diluted fractions were used for PCR analysis (S1000™ Thermal Cycler, Bio-Rad Laboratories, USA). Medicago UBQ10 was amplified for 25 cycles (94 °C for 30 s, 55 °C for 30 s, and 72 °C for 30 min).

Transcript levels of genes were measured by real-time PCR using SYBR® Green PCR Master Mix (Applied Biosystems, USA) and a CFX 96 Real-Time System (Bio-Rad Laboratories, USA). Reactions contained 5 µL of SYBR Green Master Mix reagent (Applied Biosystems, USA), 1 µL of cDNA, and 200 nM of each gene-specific primer in a final volume of 10 µL, and were subjected to 40 cycles of amplification. The primers were designed from the 3′-UTR to avoid any unspecific amplification (Table S2). The transcript levels were determined by relative quantification^32^ using Medicago UBQ10 and Medtr7g089600 as references. Gene expression was expressed as mean and standard error calculated based on three biological replicates with three technical replicates for each biological replicate.

### Microarray data processing and analysis

Twenty-two macrosclereid RNA samples underwent microarray analysis using the GeneChip Medicago Genome Array^33^. The Bioconductor R package^34^ was used for gene expression analysis. Briefly, raw microarray data in CEL files were imported into Bioconductor using the R package affy and background correction was performed using the RMA (Robust Multi-Array Average) method^35^. Genes that were differentially expressed throughout seed development were identified by ANOVA of the RMA expression values using the Limma R package^36^. Multiple testing correction was applied to the p-values of the F-statistics to adjust the false discovery rate. All genes with adjusted p-values of <0.001 were identified for further analysis. In this study, we only identified the significantly differentially expressed genes (DEGs) during MC development of *M*. *truncatula*. Hence, the genes with a fold-change of ≥3.0 were analyzed further. Furthermore, to identify the putative function of the genes exhibited significant expression changes during MC development, their sequences were BLASTN-searched againist *Medicago* genome sequences (Mt 4.0V1, http://jcvi.org/medicago/)^37^ using BLAST+ 2.6.0^38^ (E-value = 1e −10) to acquire a Genoscope ID number. The latest annotations for all Genoscope IDs and relational Network IDs, InterPro domain IDs, Gene Ontology IDs, Uniprot IDs and functions were published^37^, and were used as the reference for functional category and annotation.

### Bioinformatic analysis

Gene Ontology (GO) analysis was performed using agriGo^39^. Gene function ontology terms were characterized with Mapman^40, 41^ ontology terms and mapped into different biological pathways with tools embedded in the Mapman software^40, 41^. Gene function enrichment analysis within the detected modules was performed using the chi-square test (p-value < 0.05 after Benjamini-Hochberg correction). Transcripts found to be significantly differentially expressed during MC development were used to construct a weighted gene co-regulatory network (WGCNA) using a step-by-step method implemented in the WGCNA R package^42^. Firstly, a pairwise gene correlation matrix was calculated with a Pearson correlation analysis and transformed into a weighted matrix (called the adjacency matrix) with a scaling factor beta (β = 13) under the assumption that biological networks are scale free. Weights indicate the connection strength between gene pairs. Then, a dendrogram was generated with a hierarchical clustering method from the adjacency matrix. Consequently, modules (a cluster of genes with similar expression pattern) were identified by a dynamic tree-cut algorithm with a minimum module size of 30 genes and a high cutoff value at 0.3. The module eigengenes (first principal component) were estimated with principal component analysis (PCA). Finally, modules and their relationship to external traits were also identified using tools in this package^42^ by Pearson correlation analysis between the modules and external traits. The flavonoid pathway network in MC was generated with a hard cut-off value of |r| = 0.85, which made the network follow a roughly power-law distribution^43, 44^. Identified gene interactions were imported into Cytoscape^45^ version 3.0.2 for visualization and additional analysis.

## Results and Discussion

### Development of MC in *M*. *truncatula*

**Figure 1:**
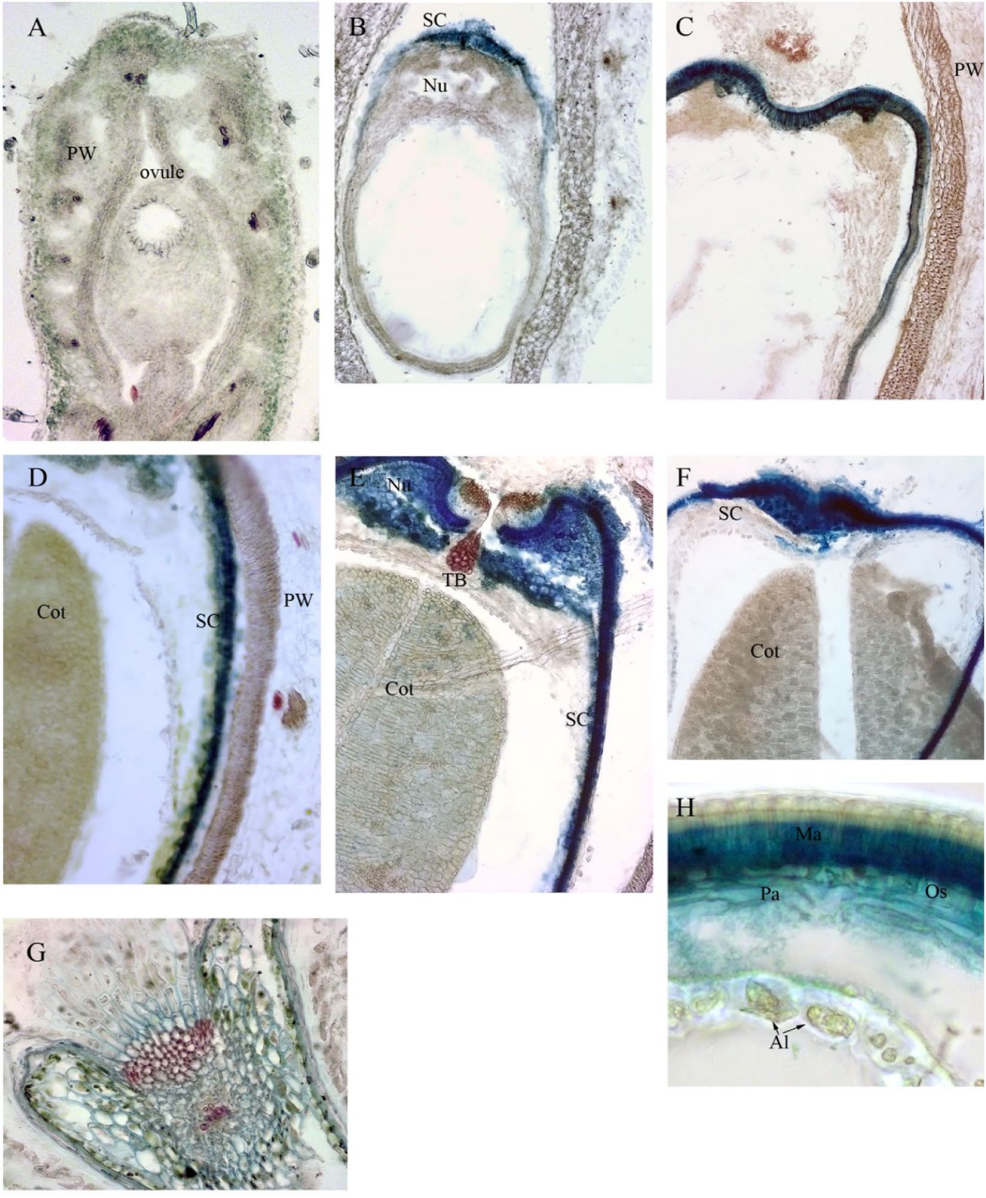
Localization of Phenolic compounds in the pods and seeds of *M*. *truncatula* at 3, 6, 13, 20, 27 and 39 DPP. (**A**) Pod at 3 DPP (134X), (**B**) Pod at 6 DPP (134X), (**C**) Pod wall, seed coat & cotyledon at 13 DPP (134X), (**D**) Pod wall, seed coat & cotyledon at 20 DPP (268X), (**E**) Pod wall, seed coat & cotyledon at 27 DPP (268X), (**F**) Pod wall, seed coat & cotyledon at 39 DPP (268X), (**G**) Wheat at 24 DPP as positive control (268X), (**H**) Seed coat at 39 DPP (538X). Al, Aleurone cell; Cot, Cotyledon; Ma, Macrosclereids; Nu, Nucellus; Ovule; Pa, Parenchyma; PW, Pod wall; SC, Seed coat; TB, Trachid bar.

**Figure 2:**
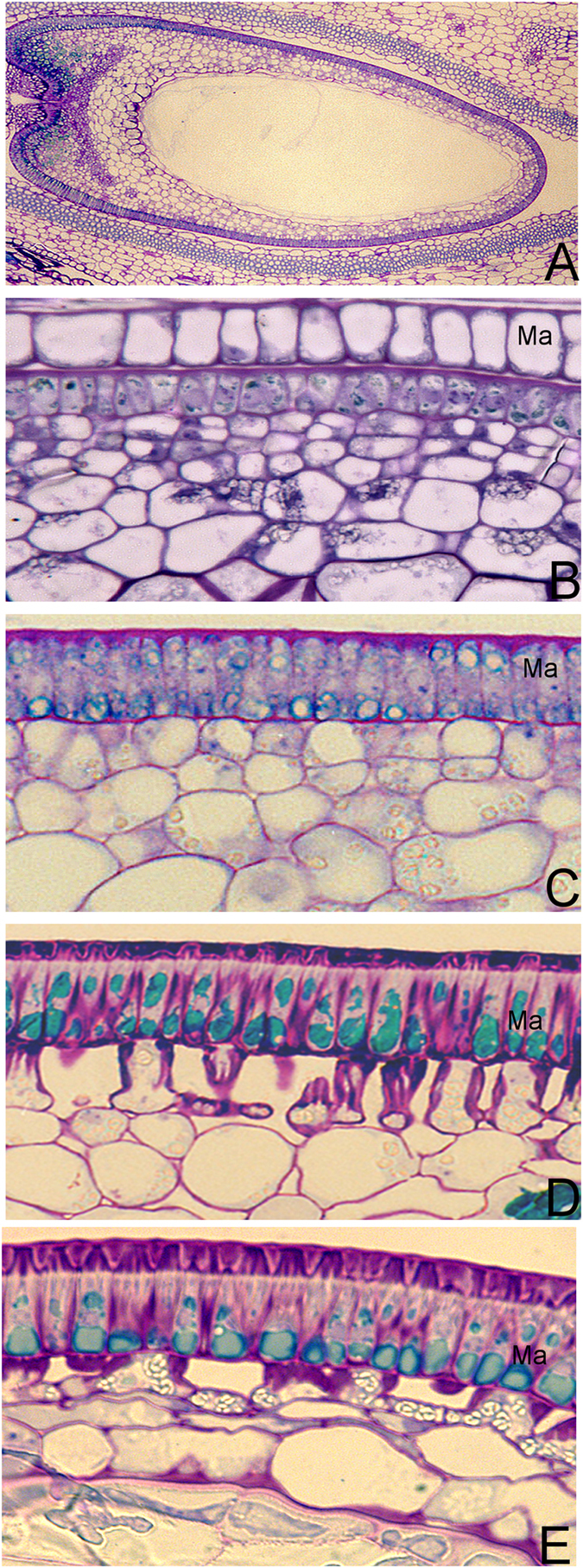
Light micrographs of developing *M*. *truncatula* MC stained with Toluidine Blue O. Ma presents MC of the seed coat. (**A**–**D**) Cross-sections through seed coats at (**A**) 3 DPP, (**B**) 6 DPP, (**C**) 13 DPP, (**D**) 20 DPP, and (**E**) 27 DPP, respectively. Phenolic compounds stain blue-green.

In previous studies, we characterized cellular structures and developmental processes for the pods and seeds of *M*. *truncatula*. Also, we investigated and described the development of MC in *M*. *truncatula* ^10^, and found polyphenolic phytochemical accumulation (e.g., flavonoids) in MC during seed developing stages of *M*. *truncatula* are changed spatially and timely. In this study, to determine the distribution of polyphenolic compounds in seeds of *M*. *truncatula*, we performed a histochemical analysis of the seeds during six developmental stages (3 DPP, 6 DPP, 13 DPP, 20 DPP, 27 DPP, and 39 DPP) using Toluidine Blue O at pH 4.4 (Fig. 1) with wheat seeds at 24 DPP as positive control. Polyphenolic compounds were stained with Toluidine Blue O showing blue-green color. No polyphenolic compounds were detected on 3 DPP (Fig. 1A). Ployphenolic compounds were accumulated at 6 DPP when seed coat starts to form (Fig. 1B). The accumulation of polyphenolic compounds progress rapidly during seed development (Fig. 1C–H). In Fig. 1, we found that polyphenolic compounds were only accumulated in the seed coat of *M*. *truncatula*. Under 500x magnification, we found that the seed coat epidermis, which will develop into MC in the later developmental stages, is the major site of phenolic compound biosynthesis and accumulation (Fig. 2). Phenolic compounds were observed in small vacuoles (Fig. 2A) of epidermis cells from the seed coats of developing *M*. *truncatula* pods at 6 DPP. Starting at 13 DPP, vacuoles were found to enlarge significantly with darker staining color observed compared to 6 DPP. These changes further intensified with seed development until maturation. Due to the observed dependence of vacuole size and the amount of polyphenolic compounds during MC development, and the fact that phenolic compounds do not accumulate in other cells of the seed coat, we considered that the cellular processes, specifically the vacuole development in MC, should be crucial to the phenolic compound metabolism of *M*. *truncatula* seed.

### Characterization of phytochemicals in MC

**Figure 3:**
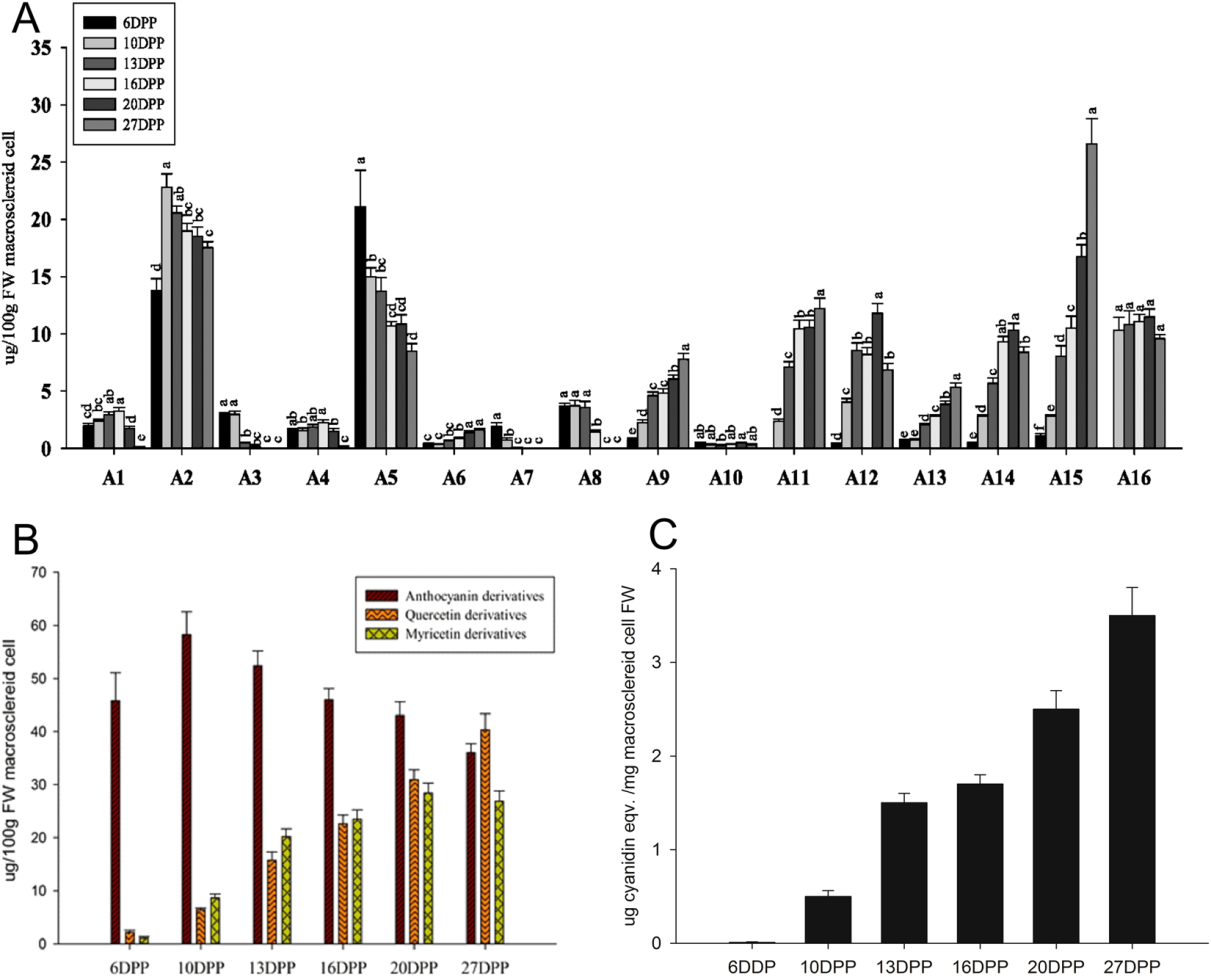
Flavonoid profiles at six different time points during *M*. *truncatula* MC development. (**A**) Sixteen flavonoids were identified by HPLC-MS using Fisher’s Least Significant Difference test (p < 0.05, LSD). The identified compounds from each profile are listed in Table 1. The HPLC conditions are described in Materials and Methods. Values represent the average ± SD of nine biological replicates. (**B**) The concentration of three types of flavonoid derivatives present in MC. Values represent the average ± SD of nine biological replicates. (**C**) In-PA content in MC using BuOH-HCl assays. Values represent the average ± SD of three biological replicates.

PA and polyphenolic compounds in *M*. *truncatula* MC samples collected at six time points of seed development were characterized using LC-MS (Fig. S1 and Table 1). We identified twenty peaks as flavonoid and flavonoid derivatives using LC-MS and UV spectrometry. According to the MS (n) fragment characteristics and the match of standard references based on our previous experiments^30^, the 16 flavonoids further confirmed in MC (Fig. 3A and Table 1), mainly were divided into three types according to flavonoid aglycones: quercetins, myricetins, and anthocyanin. Least significant difference (LSD) analysis indicated that the concentration of these flavonoids significantly changed during macrosclereid development. The concentration of anthocyanins peaked at 10 DPP. However, the concentration of quercetins and myricetins kept increasing throughout the development period and the time of peak was not observed in the current study (Fig. 3B). Furthermore, the levels of in-PAs were measured by subjecting the pellet remaining after solvent extraction directly to oxidative cleavage under hot acidic butanol. The seed coat began to accumulate in-PAs at 10 DPP (Fig. 3C). The concentration of in-PAs increased during the development of MC. The concentration of anthocyanins decreased after 10 DPP.

**Table 1:** Peak assignment for the analysis of extracts from seed coats at different development stages.

*#*	*code*	*RT* [*min*]^a^	*Max m*/*z* ^ b^	*UV* ^ d^	*Name* ^c^	*6 DPP*	*10 DPP*	*13 DPP*	*16 DPP*	*20 DPP*	*27 DPP*
1	A1	11.4	321	232, 284	Leucodelphinidin	1.97 ± 0.21^f^	2.42 ± 0.15	2.96 ± 0.23	3.26 ± 0.31	1.74 ± 0.17	0.15 ± 0.02
2, 3	A2	20.8	467	237, 293	Leucodelphinidin-glucoside	13.76 ± 1.05	22.81 ± 1.17	20.56 ± 0.61	19 ± 0.63	18.54 ± 0.79	17.54 ± 0.51
4	A3	23.3	305	225, 323	Leucodelphinidin	2.93 ± 0.17	2.97 ± 0.28	0.47 ± 0.08	0.12 ± 0.01	ND^f^	ND
5	A4	25.4	387	229, 288	Chalocone glucoside	1.66 ± 0.06	1.54 ± 0.22	1.85 ± 0.24	2.23 ± 0.28	1.5 ± 0.23	0.2 ± 0.03
6	A5	27.5	451	275	Epicatechin 3-O-glucoside	21.12 ± 3.17	15.00 ± 0.77	13.73 ± 1.18	10.7 ± 0.37	10.85 ± 0.82	8.47 ± 0.69
7	A6	29.6	449	230, 291	Luteolin 3-O-glucoside	0.36 ± 0.08	0.37 ± 0.05	0.69 ± 0.05	0.86 ± 0.09	1.37 ± 0.16	1.6 ± 0.13
8	A7	30.6	289	279	Epicatechin	1.89 ± 0.36	0.75 ± 0.17	0.09 ± 0.02	ND	ND	ND
9	A8	32.7	289	279	Catechin	3.72 ± 0.22	3.73 ± 0.48	3.55 ± 0.55	1.45 ± 0.15	ND	ND
10	A9	34.6	625	260, 350	Myricetin 3-O-rutinoside	0.76 ± 0.13	2.25 ± 0.22	4.6 ± 0.32	4.86 ± 0.36	6.06 ± 0.36	7.8 ± 0.49
11	A10	35	739	235, 277	Cindonain-(alfa-4beta-8)-catechin	0.42 ± 0.1	0.32 ± 0.08	0.25 ± 0.06	0.36 ± 0.04	0.47 ± 0.05	0.3 ± 0.08
12	A11	35.5	479	259, 357	Myricetin 3-O-galactoside	ND	2.37 ± 0.18	7.08 ± 0.49	10.45 ± 0.77	10.57 ± 0.62	12.21 ± 0.9
13, 14	A12	36.6	521	258, 357	Myricetin 3-O-galactoside-malatone	0.31 ± 0.15	4.05 ± 0.31	8.55 ± 0.65	8.2 ± 0.6	11.8 ± 0.84	6.85 ± 0.57
15	A13	36.9	610	258, 356	Rutin	0.66 ± 0.1	0.77 ± 0.07	2.06 ± 0.12	2.81 ± 0.15	3.87 ± 0.26	5.34 ± 0.36
16	A14	38.5	464	258, 354	Quercetin 3-O-Glucoside	0.42 ± 0.11	2.83 ± 0.12	5.68 ± 0.49	9.32 ± 0.45	10.33 ± 0.58	8.4 ± 0.47
17, 18	A15	39.9	505	258, 355	Quercetin 3-O-Glucoside-6″-acetone	1.12 ± 0.18	2.83 ± 0.13	8.04 ± 0.92	10.51 ± 1.04	16.73 ± 1.06	26.59 ± 2.2
19, 20	A16	41.3	467	252, 335	Epicatechin 3-O-glucuronidate	ND	10.29 ± 1.13	10.82 ± 1.2	11.09 ± 0.6	11.47 ± 0.7	9.58 ± 0.36

^a^Measured with a C^18^ Grace column and a corresponding gradient profile (see Materials and methods).

^b^Obtained with an ion trap mass spectrometer.

^c^Identified after comparison with standards and references.

^d^Obtained with a UV detector.

^e^ND, none detected; others were detected.

^f^µg/100 g fresh weight MC.

### Identification DEGs during MC development

In order to investigate the genes responsible for development of MC in *M*. *truncatula*, we perfomed microarray analysis on these samples isolated from the same plants at six time points in our LC-MS analysis. Four replicates were included for each sample point with two independent biological experiments. The expression values of 61278 probe sets were extracted from 22 chips, except that one chip of 10 DPP and 20 DPP were not successful when manipulated in GeneChip Array experiments. These biological replicates are quite consistent as shown by the fact that biological replicated samples were clustered tightly together with hierarchical cluster analysis of the 22 chips (Fig. S2). To identify the significantly changed genes, pair-wise comparisons of gene expression in the six developmental stages were performed. A total of 4681 genes were identified as significantly changes genes with an absolute fold-change of ≥3.0 and a LogRatio P-value of ≤0.001 between at least two time points during MC development (Table S4). We selected these most significantly expressed genes for subsequent analysis. To validate the microarray results, we selected twenty-five of the DEGs for independent validation using qRT-PCR (Tables S2 and S3). The trends in gene expression observed in our qRT-PCR analysis were in highly consistent with our microarray data. These results indicate that the microarray data in the present study are an accurate representation of changes in gene expression during MC development.

### Ontology (GO) classification of DEGs

**Figure 4:**
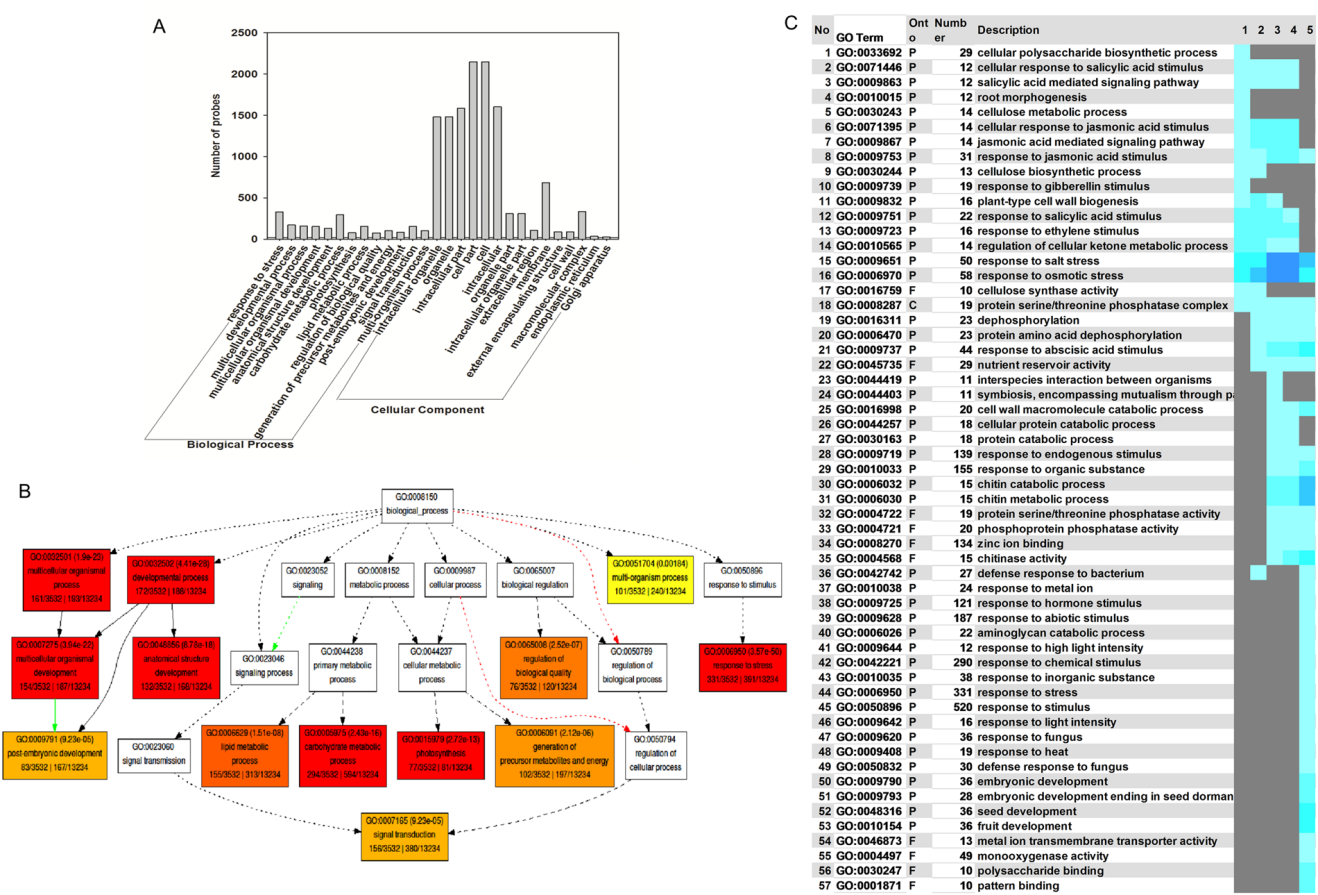
GO enrichment analysis of DEGs at six different time-points during MC development in *M*. *truncatula*. (**A**) GO analysis of DEGs. (**B**) Enrichment GO “Biological process” analysis of the DEGs. See Supplemental Fig. S3 for the “Cellular component” categories. (**C**) The down-regulated GO terms were analyzed by PAGE (Tables S5 and S6). The colored blocks represent the level of up/downregulation of each term at a certain developmental stage. The yellow-to-red, cyan-to-blue, and grayscale represent up-regulation, down-regulation, and no significant change, respectively. Detailed information of each term is provided in Table S5.

We next performed GO classification analysis based on significantly DEGs using SEA tool of agriGO^39^. 1994 and 2431 genes were assigned to two major categories (biological process and cellular component) respectively. GO terms of response to stress, developmental process, multicellular organismal process, multicellular organismal development, and anatomical structure development were predominant based on their p-values in the biological process category (Fig. 4A and B, Table S4). GO terms of intracellular organelle, organelle, intracellular part, cell part, and cell were identified as being the most abundant classes in the cellular component category (Fig. S3, Table S5). To cross-compare the gene expression levels at different time-points during MC development, the fold-change value (comparison with the corresponding value at 6 DPP) of these genes at other five time-points was used as input for PAGE (P < 0.05, FDR = 5%) (Fig. 4C, Table S6). Fifty-seven GO terms were down-regulated during MC development. Intriguingly, the genes that were down-regulated during the later developmental stages were mainly involved in the biotic or abiotic stress response, including 121 genes involved in the response to hormone stimulus, 187 genes involved in the response to abiotic stimulus, 290 genes involved in the response to chemical stimulus, 331 genes involved in the response to stress, and 520 genes involved in the response to stimulus (Table S6).

Recently, vesicle-mediated flavonoid transport was observed in a microscopy-based study. Anthocyanoplasts are thought to be transporter of anthocyanin or to serve as the sites of anthocyanin biosynthesis^14, 46–49^. In addition, anthocyanin-containing vesicle-like structures are co-localized with protein storage vacuoles (PSVs) and transport anthocyanins in a trans-Golgi network (TGN)-independent ER-to-PVC vesicle trafficking pathway^50^. It was previously reported that prevacuole-like vesicle structures containing PAs can merge with the central vacuole^46^. More recently, *Arabidopsis* mutants with reduced PA production (i.e., *tt* mutants) were found to exhibit morphological defects in the central vacuole of the seed coat endothelium cells^26, 36, 51–53^. Furthermore, most of the genes up-regulated in MC during later developmental stages were involved in compound transportation; specifically, 335, 360, 360, 360, and 360 genes were identified as being involved in the transport of the macromolecular complex, membrane-bounded vesicle, vesicle, cytoplasmic vesicle, and cytoplasmic membrane-bounded vesicle, respectively (Table S6). These results indicate the importance of vesicle-mediated flavonoid transport in *M*. *truncatula* MC.

### MC are the first barrier of defense in *M*. *truncatula* seeds

A total of 815 genes were associated with abiotic and biotic stress, such as defense, disease resistance, signaling, protein degradation, and hypersensitive response. These defense-related genes were divided into several functional categories, including the cell wall (115), secondary metabolism (87), hormone metabolism (87), stress (108), redox (38), miscellaneous (52), RNA (60), protein (146), and signaling (172) (Table S7).

Plant NBS-LRR proteins are receptors that directly or indirectly recognize pathogen-deployed proteins, and this specific reorganization triggers the plant defense response^54, 55^. We detected fourteen differentially expressed NBS-LRR proteins (p < 0.05) during MC development, including those belonging to the TIR-NBS-LRR (7 genes), leucine-rich repeat family protein (3 genes), CC-NBS-LRR (2 genes), and like LRR protein (2 genes) classes.

In addition, sixty transcription factors (TFs, p < 0.05) associated with defense resistance were differentially expressed in MC, including those belonging to the MYB domain transcription factor family (20 genes), MYB-related transcription factor family (5 genes), AP2/EREBP family, APETALA2/ethylene-responsive element binding protein family (9 genes), WRKY domain transcription factor family (6 genes), bZIP transcription factor family (15 genes), and C2C2(Zn) DOF zinc finger family (5 genes). WRKY4^56, 57^, WRKY23^58, 59^, WRKY33^60–62^, and WRKY40^63–65^ are known to be involved in the pathogen response in plants. Several WRKY factors may be involved in elicitor-triggered reprogramming of secondary metabolites in *M*. *truncatula* ^49^. The overexpression of four WRKY genes in *Nicotiana tabacum* (tobacco) demonstrated that the encoded proteins regulate lignin deposition, PR gene expression, and systemic defense responses against tobacco mosaic virus^66^. In this study, WRKY4, WRKY23, WRKY33, WRKY40, and WRKY56 were identified as significantly induced during MC development.

Finally, genes with other biological processes involved in the plant defense system including those related to the cell wall, secondary metabolism, hormone metabolism, protein, and signaling also exhibited differential expression during MC development (Table S7). Microarray analysis results indicate that genes accumulated with the highest expression during MC development, were defense-related proteins specifically related to the cell wall, secondary metabolism, hormone metabolism, stress, protein process, and defense response signaling. With the fact that MC form the outer cell layer of the seed coat, which covers the seed and protects the enclosed embryonic tissues, are the first line of defense in the *M*. *truncatula* seed.

### TFs involved in flavonoid biosynthesis during MC development

**Figure 5:**
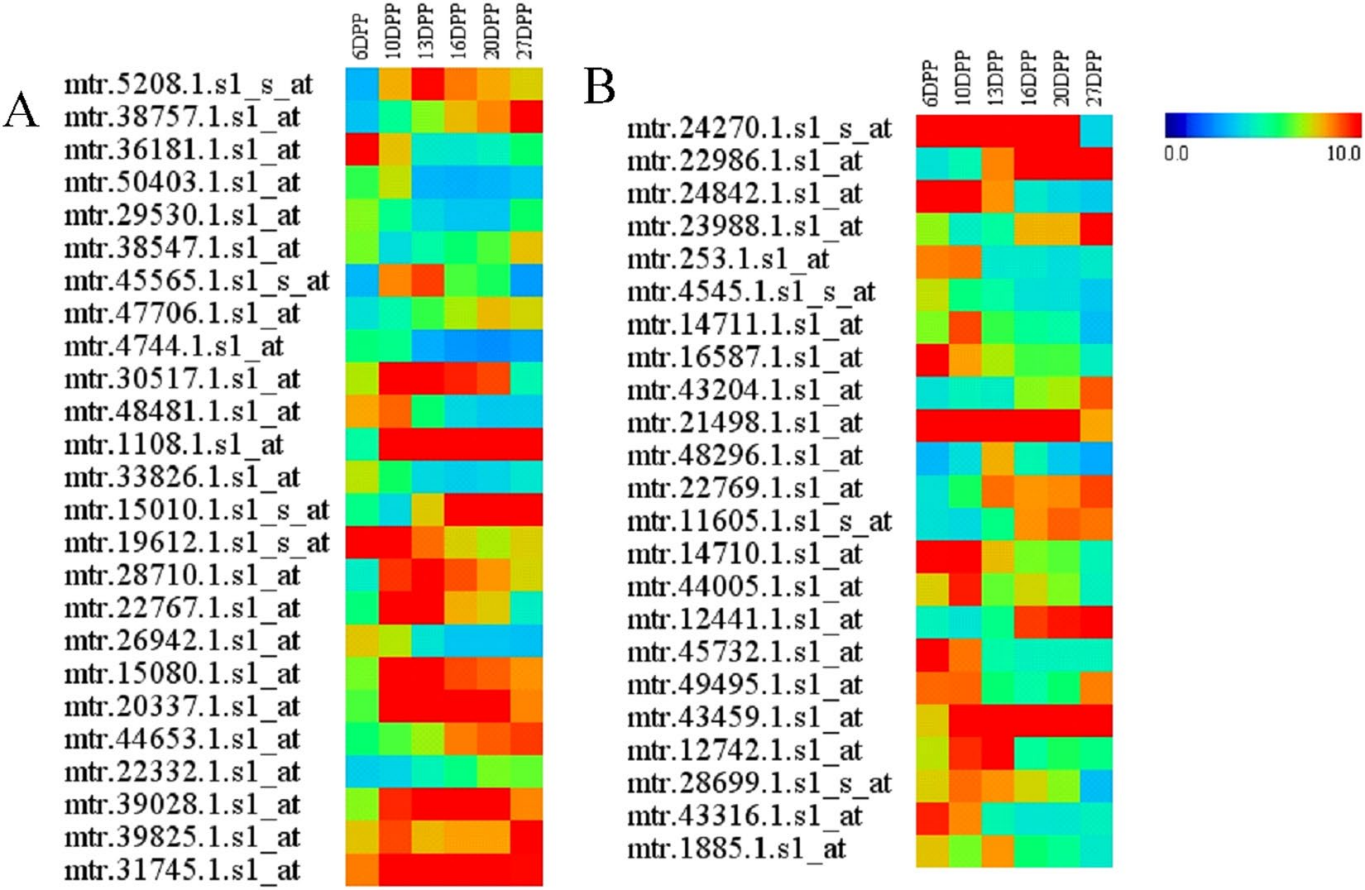
Expression profiling of MYB and bHLH families during MC development. Relative expression values were used to construct the heat map. (**A**) shows the expression profile of the MYB family. (**B**) shows the expression profile of the bHLH family. Additional information regarding the transcriptional factor genes is presented in Table S8.

A total of 223 putative TFs were significantly differentially expressed at the six time-points examined in MC, including members of the AP2/ERF, bHLH, C2H2, HB, MADS, MYB, WRKY, and bZIP families. bHLH and MYB subfamily proteins were previously shown to be involved in the accumulation of anthocyanins and related phenylpropanoids^7, 21–23, 67–70^. Among the 223 putative TFs, twenty-five MYB TFs (Fig. 5A) and twenty-three bHLH TFs (Fig. 5B) were found to be significantly differentially expressed during MC development, including *MYB3R1*, *MYB4*, *MYB16*, *MYB55*, *MYB61*, *MYB70*, *MYB77*, *MYB78*, *MYB112*, *MYB123*, *bHLH096*, *bHLH093*, *SPT*, *NAI1*, *PAP3*, *SPT*, and *ILR3* (Table S8).

MYB transcription factors, basic helix-loop-helix (bHLH) transcription factors, and tryptophan–aspartic acid repeat (WDR) proteins regulate the expression of genes involved in flavonoid biosynthesis^71, 72^. The *Arabidopsis* genome contains 133 R2R3-MYB genes^73^, whereas *M*. *truncatula* has 91 (IMGAG database). *MYB11*/*PFG2*, *MYB12*/*PFG1*, and *MYB111*/*PFG3* were reported to regulate flavonol biosynthesis in *Arabidopsis* ^74, 75^. In addition, several MYB transcription factors were identified as repressors of the flavonoid pathway, specifically of anthocyanin biosynthesis, including *FaMYB1* from strawberry (*Fragaria *× *ananasa*)^76^, *AtMYBL2*, *AtMYB4*, and *AtMYB60* from *Arabidopsis* ^77–79^, and *AmMYB308* from *Antirrhinum* ^80^.

Furthermore, plant bHLH transcription factors are important regulators of flavonoid biosynthesis. *AtTT8*, an important bHLH protein in *Arabidopsis*, was found to regulate both the anthocyanin and PA pathways^81^. In addition, a small number of WD40 proteins were identified as regulators of flavonoid biosynthesis, including petunia ANTHOCYANIN11 (*AN11*)^82^, *Arabidopsis* TRANSPARENT TESTA GLABRA 1 (*TTG1*)^20^, perilla *PFWD* (*Perilla frutescens WD repeats*)^83^, maize *ZmPAC1* (*PALE ALEURONE COLOR 1*)^84^, *M*. *trunculata MtWD40*-*1* ^7^, and *Vitis vinifera* (grapevine) *WDR1* and *WDR2* ^85^.

Most R2R3-MYB transcription factors that regulate flavonoid biosynthesis, including WDR proteins, depend on cofactors, and a small subgroup of bHLH proteins with a common motif in their N termini interact with a signature motif in the R3 repeat of the N-terminal R2R3 domain of MYB factors^86^. In *Arabidopsis*, the TT2/TT8/TTG1 complex regulates PA accumulation in the seed coat^87, 88^.

### ABC transporters are involved in MC development

**Figure 6:**
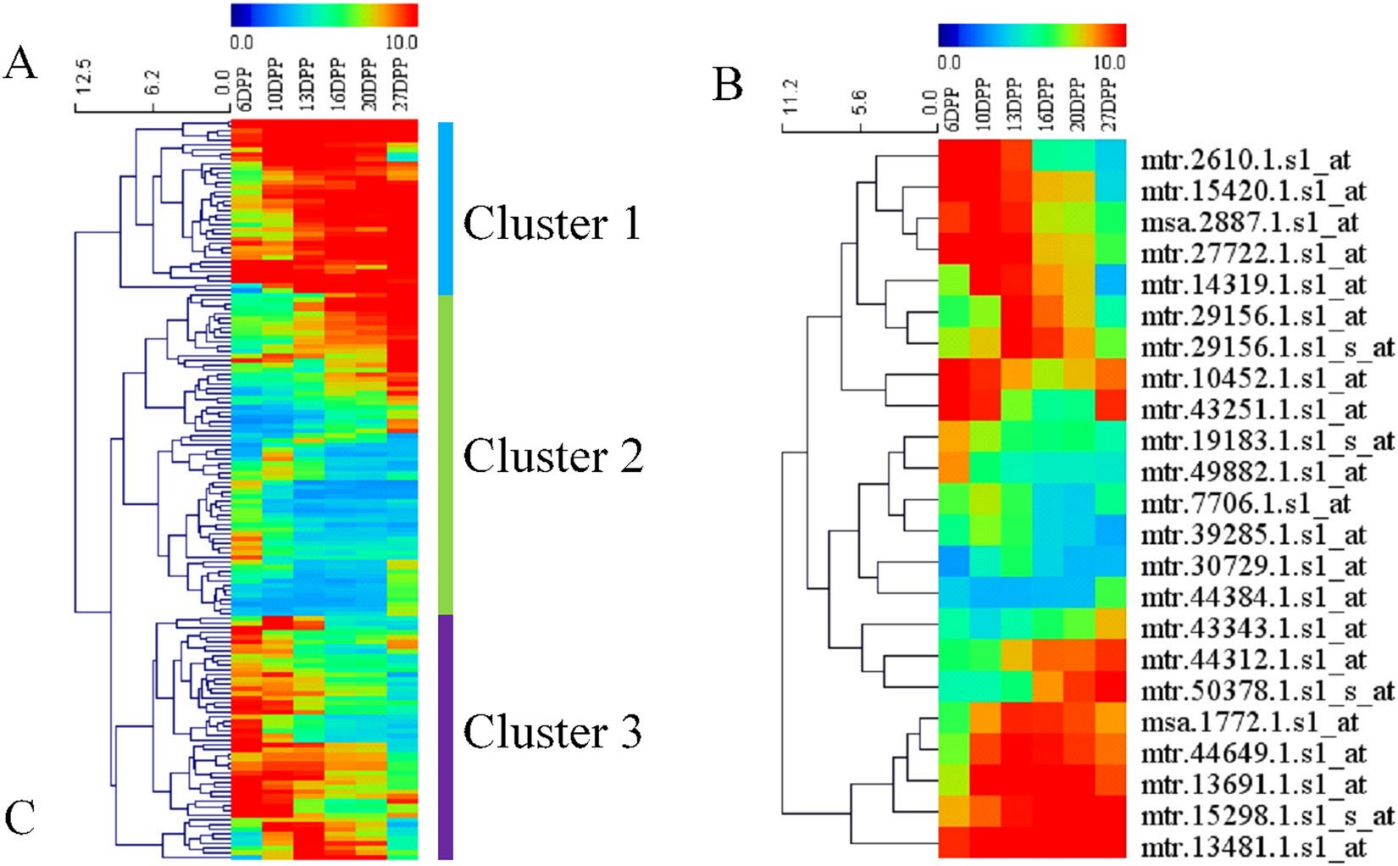
Expression profiling of transport protein genes during MC development. The relative expression values were used to construct the heat map. (**A**) Shows the expression profile of all transport protein genes. (**B**) Is the expression profile of ABC transport protein genes.

A total of 155 annotated transporter proteins showed differential expression during the development of MC tissues, including three p- and v-ATPases, 17 sugar transporters, 13 amino acid transporters, 15 peptide and oligopeptide transporters, 9 potassium transporters, 19 major intrinsic protein transporters (PIPs), and 23 ABC transporters (Fig. 6). All ABC transport proteins were grouped into three clusters by hierarchical cluster analysis with Euclidean distance (Fig. 6B). Five, eight, and ten ABC transport proteins were grouped into Cluster 1, Cluster 2, and Cluster 3, respectively. The expression level of Cluster 1 in the early developmental stages (6 DPP, 10 DPP, and 13 DPP) was higher than at other stages. By contrast, the expression level of proteins in Cluster 3, including PGP2, PDR13, MRP14, PED3, and PGP2, was up-regulated later in development.

The ABC transporter family transports a range of substrates, including metal ions, auxin, malate, defensive secondary metabolites, and xenobiotics^89^, across membranes using the energy released from ATP hydrolysis^90^. Over 120 putative ABC transporters have been identified in the *Arabidopsis*, rice (*Oryza sativa*), tomato (*Solanum lycopersicum*), and *M*. *truncatula* genomes; however, only a few of these have been characterized. MRP-type ABC transporters have been verified to be involved in flavonoid transport^91^, functioning in the long-distance transport of flavonoids, such as naringenin, dihydrokaempferol, and dihydroquercetin, via unidirectional cell-to-cell movement between the root and shoot^92^. Some multidrug and toxic compound extrusion (MATE)-type transporters have been implicated in the transport and detoxification of xenobiotics and a wide range of metabolites, such as cations, organic acids, and secondary metabolites. *MATE1* was identified as preferentially transporting epicatechin 3′-O-glucoside, a precursor for proanthocyanidin biosynthesis. *MATE1* complements the seed proanthocyanidin phenotype of the *Arabidopsis tt12* mutant both quantitatively and qualitatively. On the basis of biochemical properties, tissue-specific expression pattern, and genetic loss-of-function analysis, *MATE1* has been identified as an essential membrane transporter for proanthocyanidin biosynthesis in the *Medicago* seed coat. Subsequently, *MATE2* has also been considered to act as a membrane transporter during proanthocyanidin biosynthesis in *Medicago* leaves^9^. In our study, a total of 155 annotated transporter proteins showed differential expression during the development of MC tissues, including 23 ABC transporters. These results indicate that transporter proteins transport a variety of substrates, including flavonoids. Furthermore, they offer a clue as to how other flavonoids, such as quercetin and myricetin, are transported into the vacuole.

### Characterization of metabolism during MC development

Using the ‘Metabolism overview’ pathway in MapMan, we identified significant transcriptional changes in genes with putative functions in metabolism (Fig. S4). Genes related to primary and secondary metabolic processes, including minor and major carbohydrate metabolism; cell wall biosynthetic processes; energy-generating processes; amino acid, lipid, wax, terpene, and flavonoid biosynthetic processes; phenylpropanoid and phenolic processes; and nucleotide metabolism account for 10.53% of all transcriptional changes. Our results show that metabolism is one of the dominant biological processes in MC development. Further analysis of 4680 genes using the Kyoto Encyclopedia of Genes and Genomics (KEGG)^93–96^ showed that 1028 genes were assigned to 294 ECs with 103 biosynthesis pathways (Table S9). In this study, we focused on the “biosynthesis of secondary metabolites” pathway in relation to flavonoid biosynthesis based on our HPLC-MS results and MC structure analysis. We identified 102 genes as being involved in flavonoid biosynthesis, phenylpropanoid biosynthesis, and flavone and flavonol biosynthesis (Table S10), including ten genes representing genes encoding enzymes that were enriched in flavonoid biosynthesis (P < 0.001). We reconstructed the flavonoid biosynthesis pathway using the KEGG database. These results indicate that most of the enzymes in the flavonoid biosynthesis pathway could be identified among the DEGs in MC (Fig. S5). There were significantly differentially expressions of the genes encoding Flavonoid 3′,5′-hydroxylase (F3′5′H), Dihydroflavonol-4-reductase (DFR, TT3), Leucoanthocyanidin dioxygenase (LDOX), Flavonol synthase/flavanone 3-hydroxylase (FLS), Leucoanthocyanidin reductase (LAR), Chalcone synthase, Caffeoyl-CoA O-methyltransferase (COMT), and 4-coumarate-CoA ligase (4CL) in MC development (Table S10).

### Building the model system for regulatory network of flavonoid biosynthesis in MC

**Figure 7:**
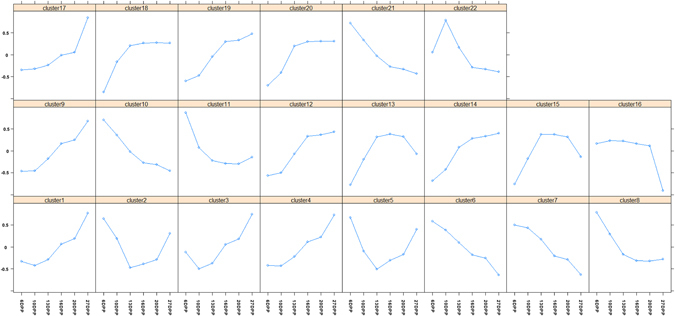
Clustering of the expression profiles of 2110 differentially expressed probes at six different time-points during *M*. *truncatula* MC development. Clustering was performed using k-means statistics and 22 clusters were chosen for further analysis of transcriptional patterns. The number of genes in each cluster is listed in parentheses. The X-axis indicates the number of days post pollination (DPP). The Y-axis indicates the LOG_2_-transformed fold-change of stage-specific intensity relative to the baseline intensity of each gene.

**Figure 8:**
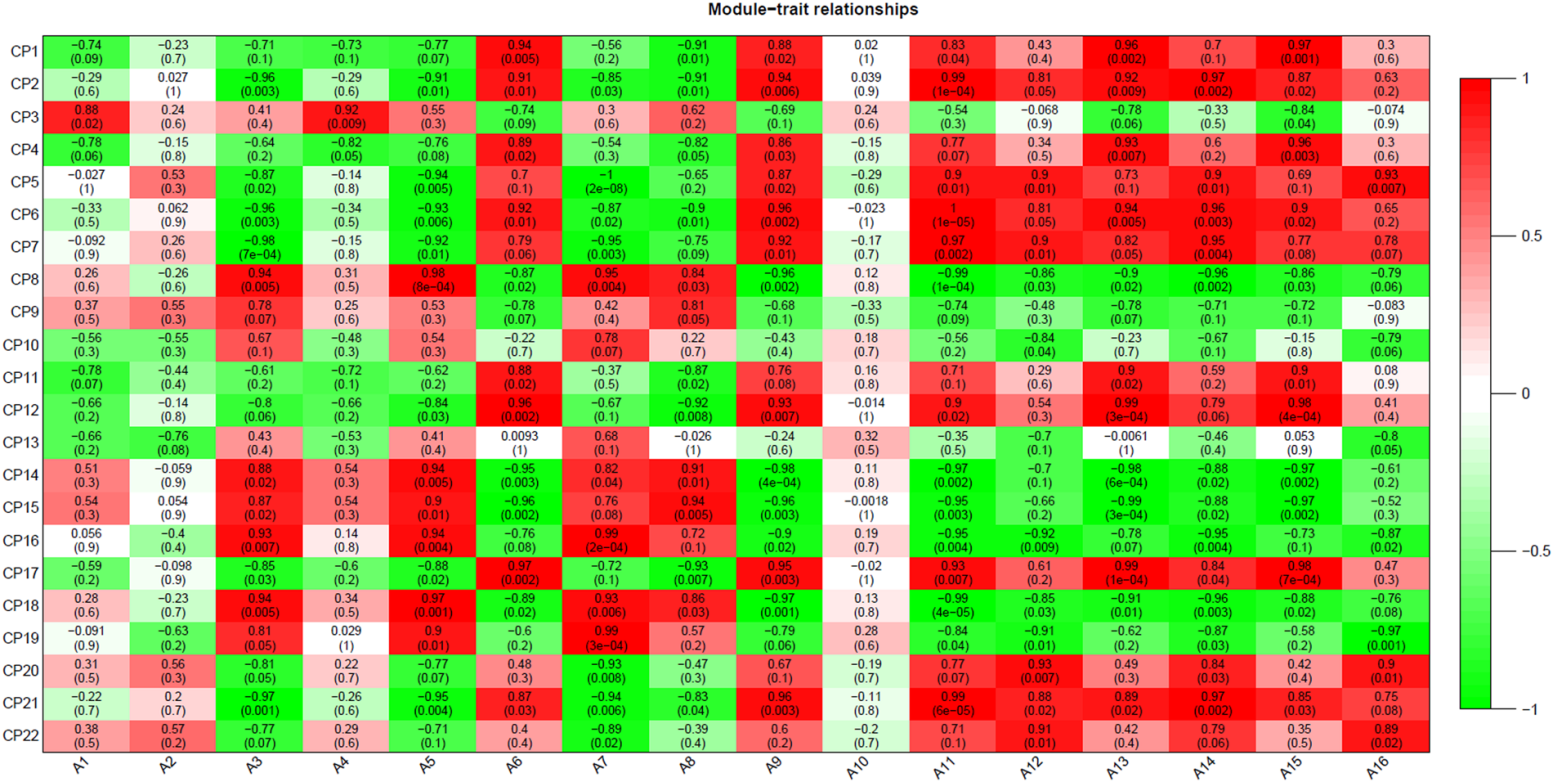
Correlation analysis between *K*-means clusters and concentration of identified flavonoids during *M*. *trunctula* MC development. Correlation between modules and flavonoid concentration is presented by colors ranging from red (highly positive correlation) to green (highly negative correlation). Columns represent the flavonoid concentration during development of MC and rows represent the modules. The correlation coefficient and p-value are shown in each cell. CP indicates the cluster number.

**Figure 9:**
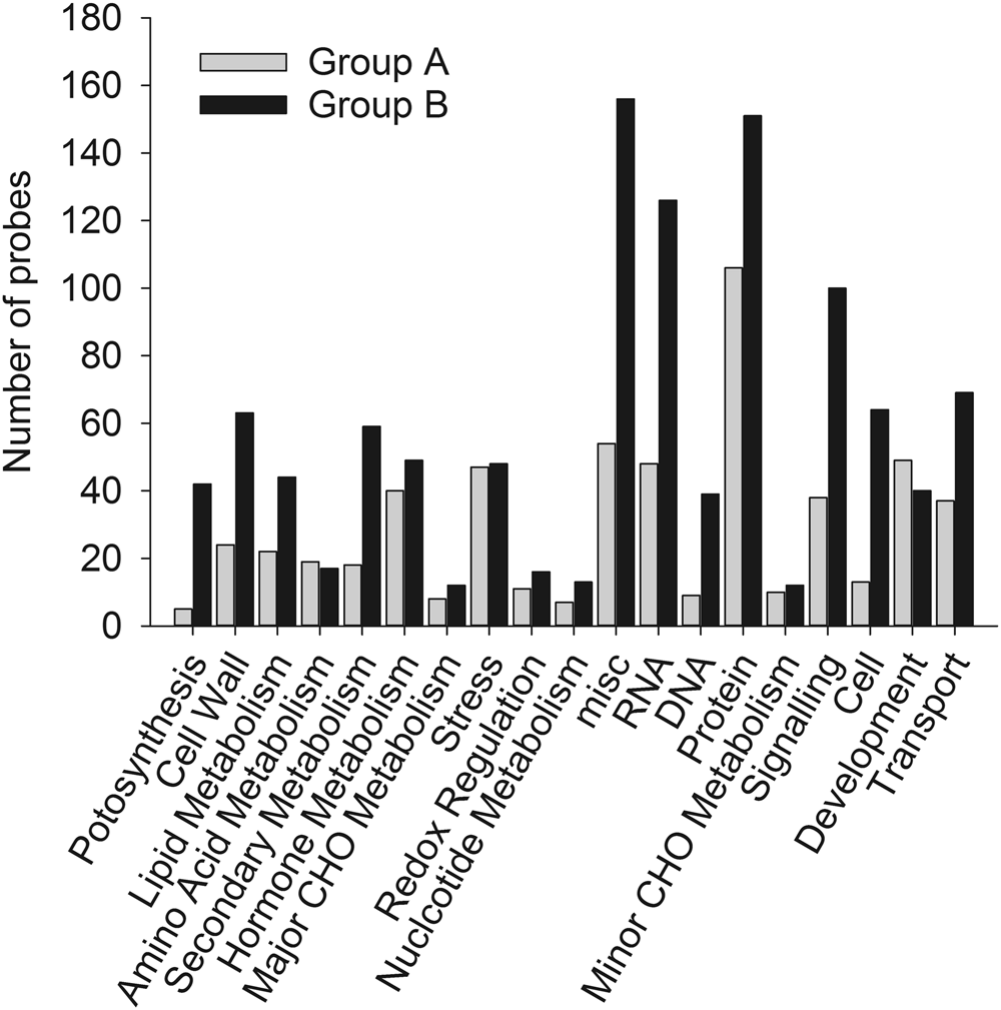
DEGs were assigned to 35 bins in the ‘Overview’ visualization pathway of MapMan using Affymetrix mapping for Medicago. Black bars represent clusters CP8, 14, 15, 16, 18, and 19. White bars represent clusters CP1, 2, 4, 5, 6, 7, 11, 12, 17, 20, 21, and 22.

The 2110 genes identified based on functional category analysis using MapMan 3.0 was clustered by the *k*-means method with Euclidean distance. A total of 22 clusters were defined from these genes based on the figure of merit value (Fig. 7). These 22 clusters were divided into four groups. Probes in the first group, including Cluster 3, 8, 14, 15, 16, 18, and 19, were down-regulated throughout MC development. These clusters contained a total of 715 genes. The second group representing clusters of genes including Cluster 9 (88 genes), 20 (76 genes), and 22 (46 genes) were first up-regulated and then down-regulated during MC development. The third group, consisting of clusters of genes including Cluster 10 (62 genes), 11 (93 genes), and 13 (71 genes), were first down-regulated and then up-regulated during MC development. Clusters of genes in the fourth group (883 genes, split up between clusters 1, 2, 4, 5, 6, 7, 12, 17, and 21) were up-regulated during MC development. We analyzed the relationship between gene expression level and flavonoid accumulation during MC development by constructing a weighted gene co-regulatory network (WGCNA) (Fig. 8). Four flavonoids, i.e., A3 (leucodelphinidin), A5 (epicatechin 3-O-glucoside), A7 (epicatechin), and A8 (catechin), were significantly positively associated with six clusters (Group A: CP8, 14, 15, 16, 18, and 19, total of 603 genes) and negatively related to 12 other clusters (Group B: CP1, 2, 4, 5, 6, 7, 11, 12, 17, 20, 21, and 22, total of 1177 genes). Eight flavonoids, i.e., A6 (luteolin 3-O-glucoside), A12 (myricetin 3-O-galactoside-malatone), A13 (rutin), A15 (quercetin 3-O-Glucoside-6″-acetone), A9 (myricetin 3-O-rutinoside), A16 (epicatechin 3-O-glucuronidate), A14 (quercetin 3-O-Glucoside), and A11 (myricetin 3-O-galactoside), were positively related to 12 clusters (Group B: CP1, 2, 4, 5, 6, 7, 11, 12, 17, 20, 21, and 22) and negatively related to 6 other clusters (Group A: CP8, 14, 15, 16, 18, and 19). A1 (leucodelphinidin), A2 (leucodelphinidin-glucoside), A4 (leucodelphinidin), and A10 (cindonain-(α-4β-8)-catechin) were not significantly related to any cluster. These results indicate that Flavan-3-ol and flavonol accumulated during MC development. Flavan-3-ol accumulated in the early developmental stages, whereas flavonol accumulated in the late developmental stages. Except for the genes in the functional category “development”, which were more abundant in Group A than in Group B, more genes in Group B were linked to functional categories than in Group A (Fig. 9).

**Figure 10:**
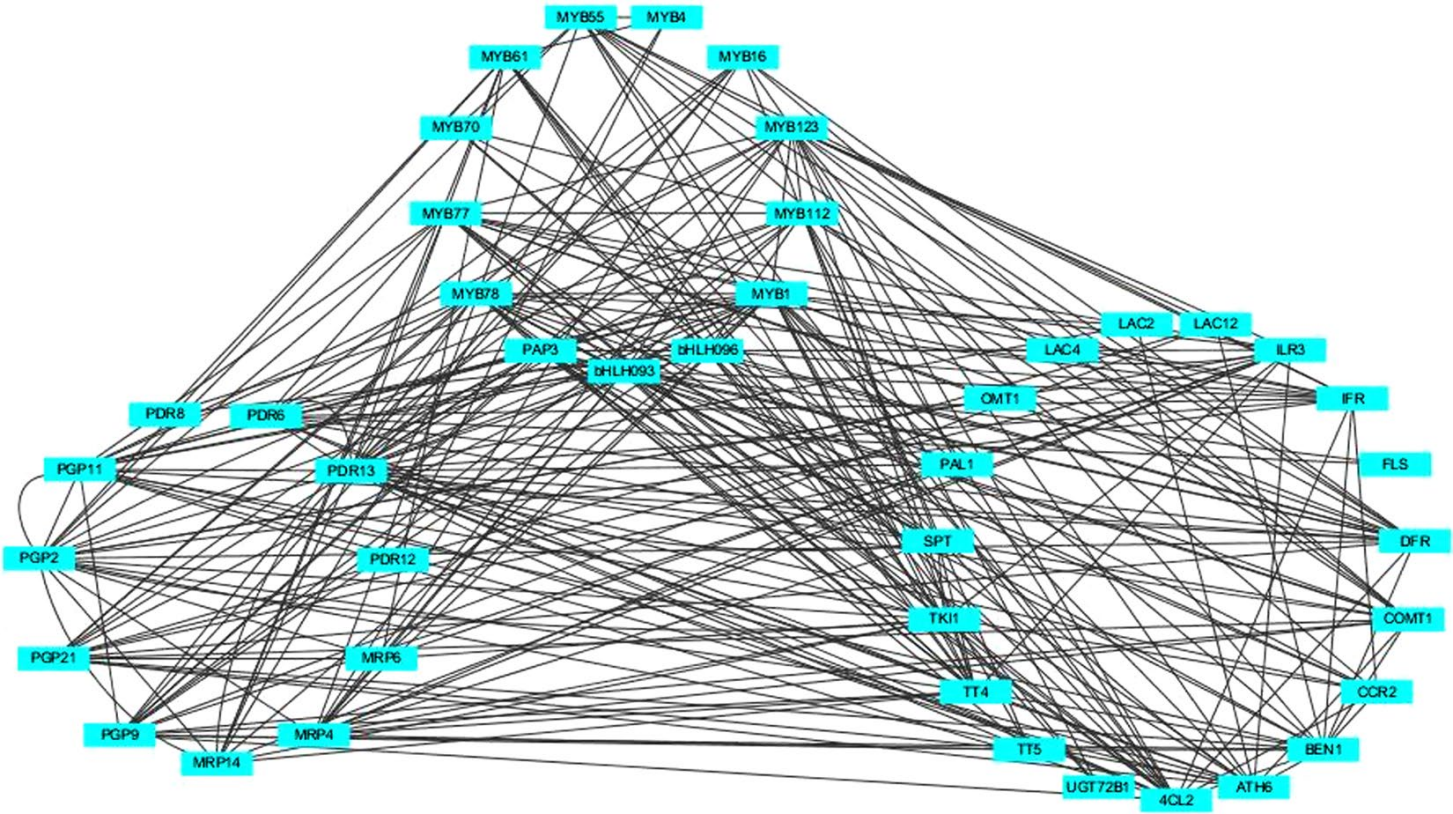
The flavonoid pathway network in MC with a hard cut-off value of |r| = 0.85 using Cytoscape. Nodes indicate individual genes, and edges indicate two genes that are co-expressed, with a cut-off value of |r| = 0.85.

We identified 19 flavonoid pathway related genes, including 11 transporter protein genes and 13 transcription factor genes amongst the 2110 genes. To investigate the interaction between these genes, we constructed a network for MC based on a hard cut-off value of |r| = 0.85 using Cytoscape (Fig. 10). The network showed co-expression of transcription factors with transporter proteins and flavonoid biosynthesis genes. Furthermore, genes encoding transporter proteins were co-expressed with the flavonoid biosynthesis related genes. These results indicated that transcription factors and transporter proteins are involved in flavonoid biosynthesis in MC development. In addition, transporter proteins may transport flavonoids during MC development.

**Figure 11:**
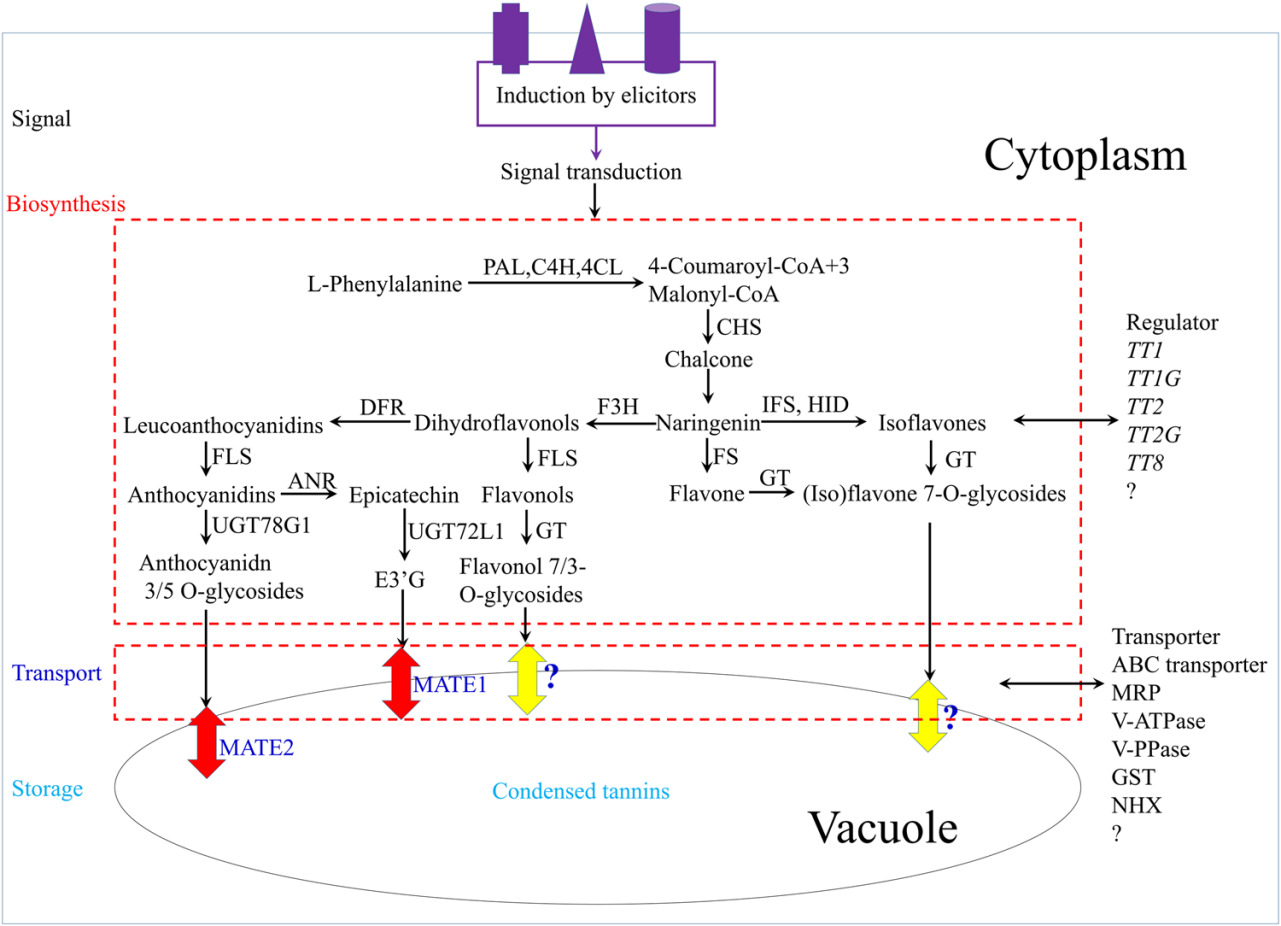
A model of potential flavonoid and isoflavonoid metabolic processes in *M*. *truncatula*. PAL, L-Phe ammonia-lyase; C4H, cinnamate 4-hydroxylase; 4CL, 4-coumarate:CoA ligase; CHS, chalcone synthase; CHI, chalcone isomerase; F3H, flavanone 3-hydroxylase; DFR, dihydroflavonol reductase; FS, flavone synthase; IFS, isoflavone synthase; HID, 2-hydroxyisoflavanone dehydratase; FLS, flavonol synthase; ANS, anthocyanidin synthase; ANR, anthocyanidin reductase; GT, glycosyltransferase; MaT, malonyl CoA:flavonoid acyltransferase; MATE, multidrug and toxin extrusion transporter; V-ATPase, vacuolar ATPase; GST, glutathione S-transferases; NHX, H/Na exchanger.

Based on the WGCNA network results, flavonoid biosynthesis can be divided into four stages in the MC of *M*. *truncatula* (Fig. 11). In the early stages of development of MC, various signals activate flavonoid biosynthesis. Only small amounts of flavonoids were detected at 6 DPP and genes related to flavonoid biosynthesis were expressed at lower levels at 6 DPP than at other stages. After 6 DPP, genes of the flavonoid biosynthesis pathway are activated, and large amounts of flavonoids are biosynthesized and modified in the cytoplasm of MC. Numerous novel flavonoids, isoflavonoids, and related biosynthetic genes have been identified and characterized via metabolomic and genomic analyses, including *TT3*, *TT4*, *TT5*, *TT6*, and *TT7*. In addition, several TFs that regulate flavonoid biosynthesis, such as *TT1* ^47^, *TTG1* ^20^, *TT2* ^21^, *TTG2* ^22^, and *TT8* ^23^ have been reported. In our gene expression profiles, 223 genes encoding putative TFs exhibited significantly differentiated expression during MC development. This finding suggests that a large number of TFs are involved in the development of MC. However, all flavonoids produced need to be transported to and stored in the vacuole via various kinds of transporters.

TT12^25^, TT19^26^, MATE1^8^, and MATE2^9^ were previously reported to function as transporters of E3′G and Cy3G. However, we identified large amounts of quercetin and myricetin derivatives in MC. Although it is unclear how quercetin and myricetin are transported into the vacuole, we found that 155 annotated transporter proteins, including three p- and v-ATPases and twenty-three ABC transporters, were differentially expressed during MC development. This finding indicates that other transporters may transport flavonoids in MC. Finally, all flavonoids were polymerized and stored in the vacuole as procyanidin oligomers, anthocyanin complexes, or other polyphenols. Thus, our results show that *M*. *truncatula* MC are an excellent model system in which to study flavonoid biosynthesis.

## Electronic supplementary material

Supplemental Figures

Supplementary Dataset 10
