# Supplementary material for: Establishment of the model system between phytochemicals and gene expression profiles in Macrosclereid cells of Medicago truncatula
Source: Sci Rep. 2017 May 31;7:2580. doi: 10.1038/s41598-017-02827-5 (PMC5451464; doi:10.1038/s41598-017-02827-5)
Supplement: Supplementary file 1 — Supplemental Figures [file 41598_2017_2827_MOESM1_ESM.pdf]

Establishment of the model system between phytochemicals and gene expression profiles in Macrosclereid cells of *Medicago truncatula*

Fuyou Fu[†], Wentao Zhang[€], Yuan-Yuan Li[$], Hong Li Wang[†*]

† Biology Department University of Arkansas at Little Rock, 2801 South University Ave. Little Rock, Arkansas 72204

[€] Aquatic and Crop Resources Development, National Research Council of Canada, 110 Gymnasium Place, Saskatoon, SK, S7N 0W9

[$]Research Group for Bioactive Products, Department of Biology and Chemistry, City University of Hong Kong, Kowloon, Hong Kong SAR, China

Supplemeantal Figures

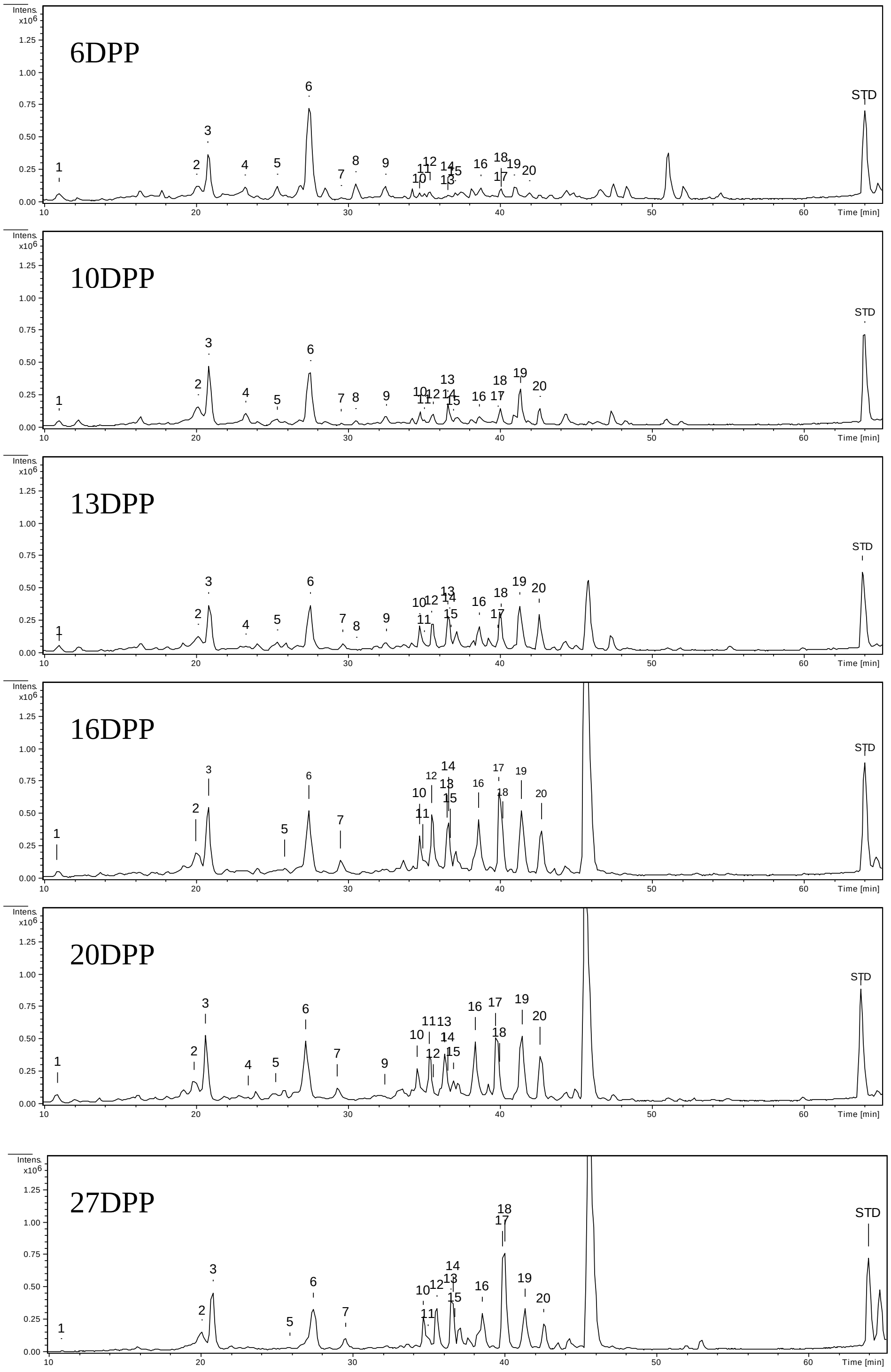

Supplemental Figure S1. LC-ESI-MS chromatograms of the extract from different development stages seed coat. Peak assignments are listed in Table 1.

Hierarchical cluster analysis of 22 microarray chips

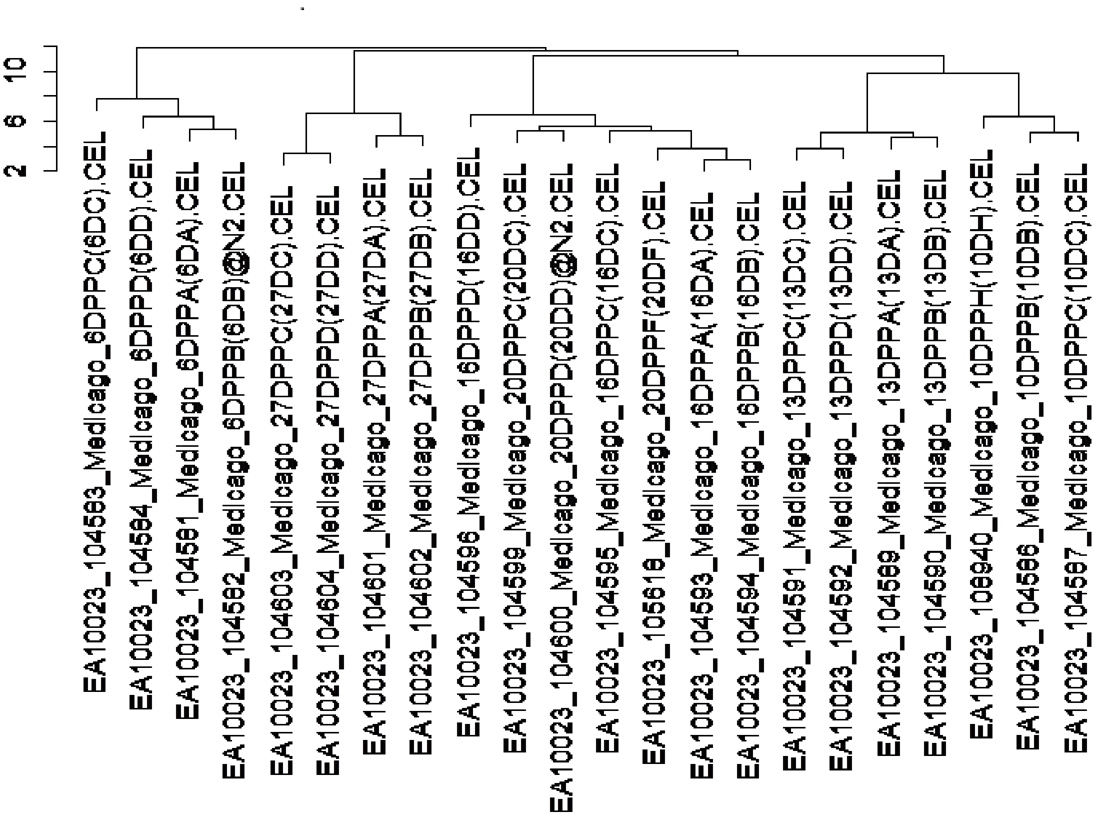

Supplemental Figure S2. Hierarchical cluster analysis of all probes in 22 microarray chips prepared from samples harvested at six time-points in *M. truncatula* macrosclereid cell development. Distance was presented by Euclidean distance with average linkage.

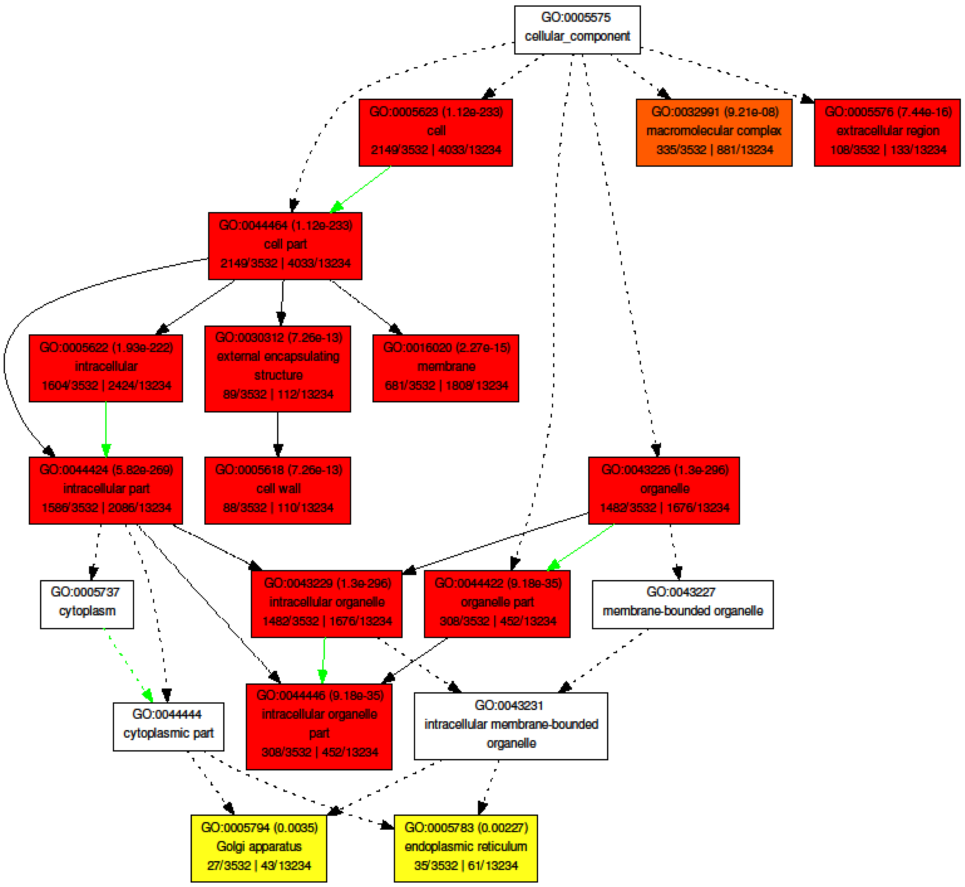

Supplemental Figure S3. Enrichment GO “Cellular component” analysis of the differentially expressed genes. Detailed information of each term is provided in Table S4. Red represents a p-value of <0.001, yellow represents a p-value >0.001, and white represents no significant GO term enrichment.

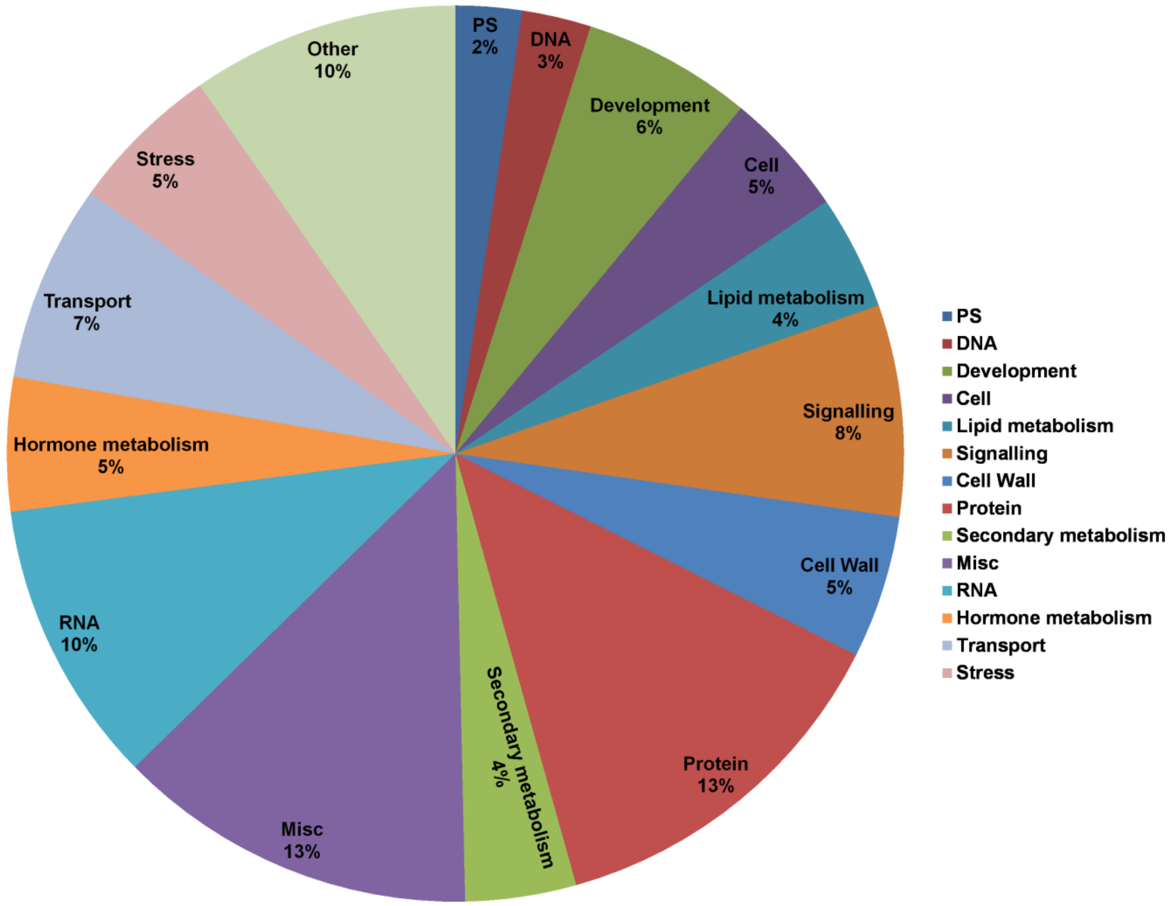

Supplemental Figure S4 Microarray analysis of samples taken at six time-points during macrosclereid cell development revealed 4681 probe sets that exhibited at least a 3-fold change in expression ($p<0.05$) using four biological replicates at each stage. Differentially expressed genes were assigned into 35 bins in the 'Overview' visualization pathway of MapMan using Affymetrix mapping for Medicago. The frequency of each bin is reported as a percentage (%) of the total number of mapped changes (4681). The details were shown in Table S6.

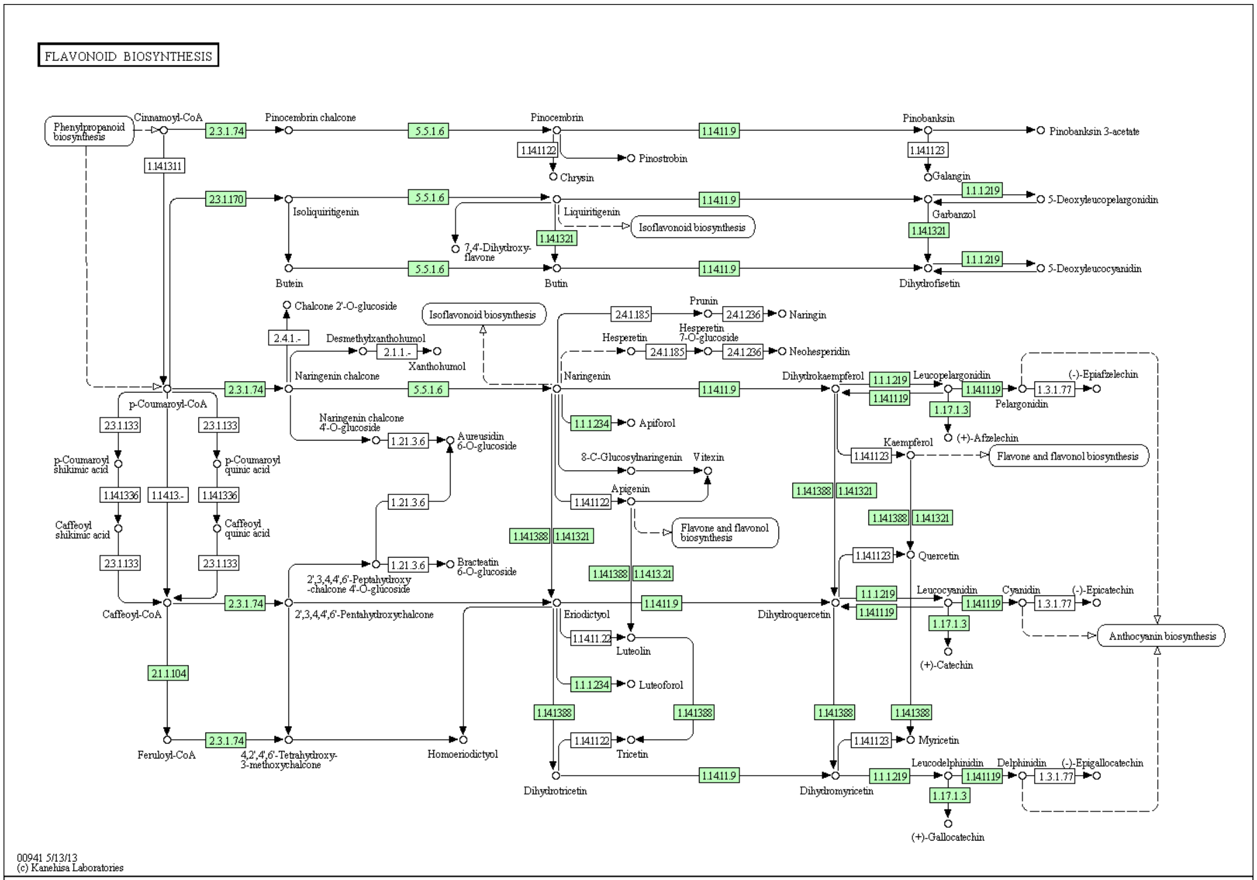
FLAVONOID BIOSYNTHESIS
Phenylpropanoid biosynthesis
Cinnamoyl-CoA
2.3.1.74
Pinocembrin chalcone
5.5.1.6
Pinocembrin
1.14.11.9
Pinobanksin
Pinobanksin 3-acetate
1.14.13.11
1.14.11.22
Chrysin
Pinostrobin
1.14.11.23
Galangin
2.3.1.170
Isoliquiritigenin
Liquiritigenin
Isoflavonoid biosynthesis
7,4'-Dihydroxy-flavone
1.14.13.21
1.1.1.219
5-Deoxyleucopelargonidin
Garbanzol
Butein
Butin
Dihydrofisetin
5-Deoxyleucocyanidin
Chalcone 2'-O-glucoside
2.4.1.-
Desmethylxanthohumol
2.1.1.-
Xanthohumol
Prunin
2.4.1.185
2.4.1.236
Naringin
Hesperetin
Hesperetin 7-O-glucoside
Neohesperidin
p-Coumaroyl-CoA
Naringenin chalcone
Naringenin
Dihydrokaempferol
Leucopelargonidin
1.14.11.19
Pelargonidin
1.3.1.77
(-)-Epiafzelechin
1.17.1.3
(+)-Afzelechin
2.3.1.133
p-Coumaroyl shikimic acid
p-Coumaroyl quinic acid
Naringenin chalcone 4'-O-glucoside
1.21.3.6
Aureusidin 6-O-glucoside
1.1.1.234
Apiforol
Kaempferol
Flavone and flavonol biosynthesis
8-C-Glucosylnaringenin
Vitexin
1.14.13.36
1.14.13.-
Caffeoyl shikimic acid
Caffeoyl quinic acid
Apigenin
1.14.13.88
Quercetin
2',3,4,4',6'-Peptahydroxy-chalcone 4'-O-glucoside
Bracteatin 6-O-glucoside
Leucocyanidin
Cyanidin
(-)-Epicatechin
Caffeoyl-CoA
2',3,4,4',6'-Pentahydroxychalcone
Eriodictyol
Dihydroquercetin
Anthocyanin biosynthesis
Luteolin
(+)-Catechin
2.1.1.104
Luteoforol
Myricetin
Feruloyl-CoA
4,2',4',6'-Tetrahydroxy-3-methoxychalcone
Homoeriodictyol
Tricetin
Dihydrotricetin
Dihydromyricetin
Leucodelphinidin
Delphinidin
(-)-Epigallocatechin
(+)-Gallocatechin
00941 5/13/13
(c) Kanehisa Laboratories

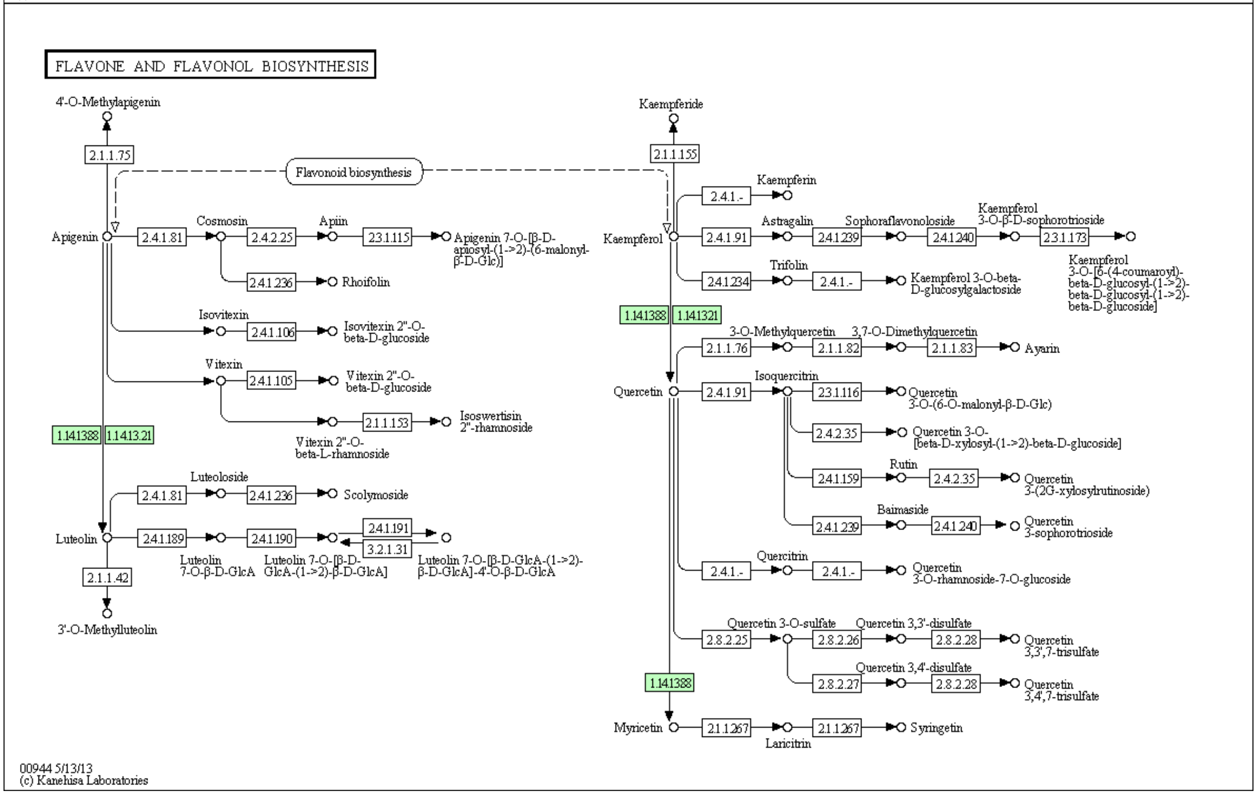
FLAVONE AND FLAVONOL BIOSYNTHESIS
4'-O-Methylapigenin
2.1.1.75
Flavonoid biosynthesis
Kaempferide
2.1.1.155
Apigenin
2.4.1.81
Cosmosin
2.4.2.25
Apiin
2.3.1.115
Apigenin 7-O-[β-D-apiosyl-(1->2)-(6-malonyl-β-D-Glc)]
2.4.1.236
Rhoifolin
Isovitexin
2.4.1.106
Isovitexin 2''-O-beta-D-glucoside
Vitexin
2.4.1.105
Vitexin 2''-O-beta-D-glucoside
2.1.1.153
Isoswertisin 2''-rhamnoside
Vitexin 2''-O-beta-L-rhamnoside
1.14.13.88
1.14.13.21
Luteoloside
Scolymoside
Luteolin
2.4.1.189
2.4.1.190
2.4.1.191
3.2.1.31
Luteolin 7-O-β-D-GlcA
Luteolin 7-O-[β-D-GlcA-(1->2)-β-D-GlcA]
Luteolin 7-O-[β-D-GlcA-(1->2)-β-D-GlcA]-4'-O-β-D-GlcA
2.1.1.42
3'-O-Methylluteolin
Kaempferol
2.4.1.-
Kaempferin
2.4.1.91
Astragalin
2.4.1.239
Sophoraflavonoloside
2.4.1.240
Kaempferol 3-O-β-D-sophorotrioside
2.3.1.173
Kaempferol 3-O-[6-(4-coumaroyl)-beta-D-glucosyl-(1->2)-beta-D-glucosyl-(1->2)-beta-D-glucoside]
2.4.1.234
Trifolin
Kaempferol 3-O-beta-D-glucosylgalactoside
1.14.13.21
3-O-Methylquercetin
3,7-O-Dimethylquercetin
2.1.1.76
2.1.1.82
2.1.1.83
Ayarin
Quercetin
Isoquercitrin
2.3.1.116
Quercetin 3-O-(6-O-malonyl-β-D-Glc)
2.4.2.35
Quercetin 3-O-[beta-D-xylosyl-(1->2)-beta-D-glucoside]
Rutin
2.4.1.159
Quercetin 3-(2G-xylosylrutinoside)
Baimaside
Quercetin 3-sophorotrioside
Quercitrin
Quercetin 3-O-rhamnoside-7-O-glucoside
Quercetin 3-O-sulfate
Quercetin 3,3'-disulfate
2.8.2.25
2.8.2.26
2.8.2.28
Quercetin 3,3',7-trisulfate
Quercetin 3,4'-disulfate
2.8.2.27
Quercetin 3,4',7-trisulfate
Myricetin
2.1.1.267
Laricitrin
Syringetin
00944 5/13/13
(c) Kanehisa Laboratories

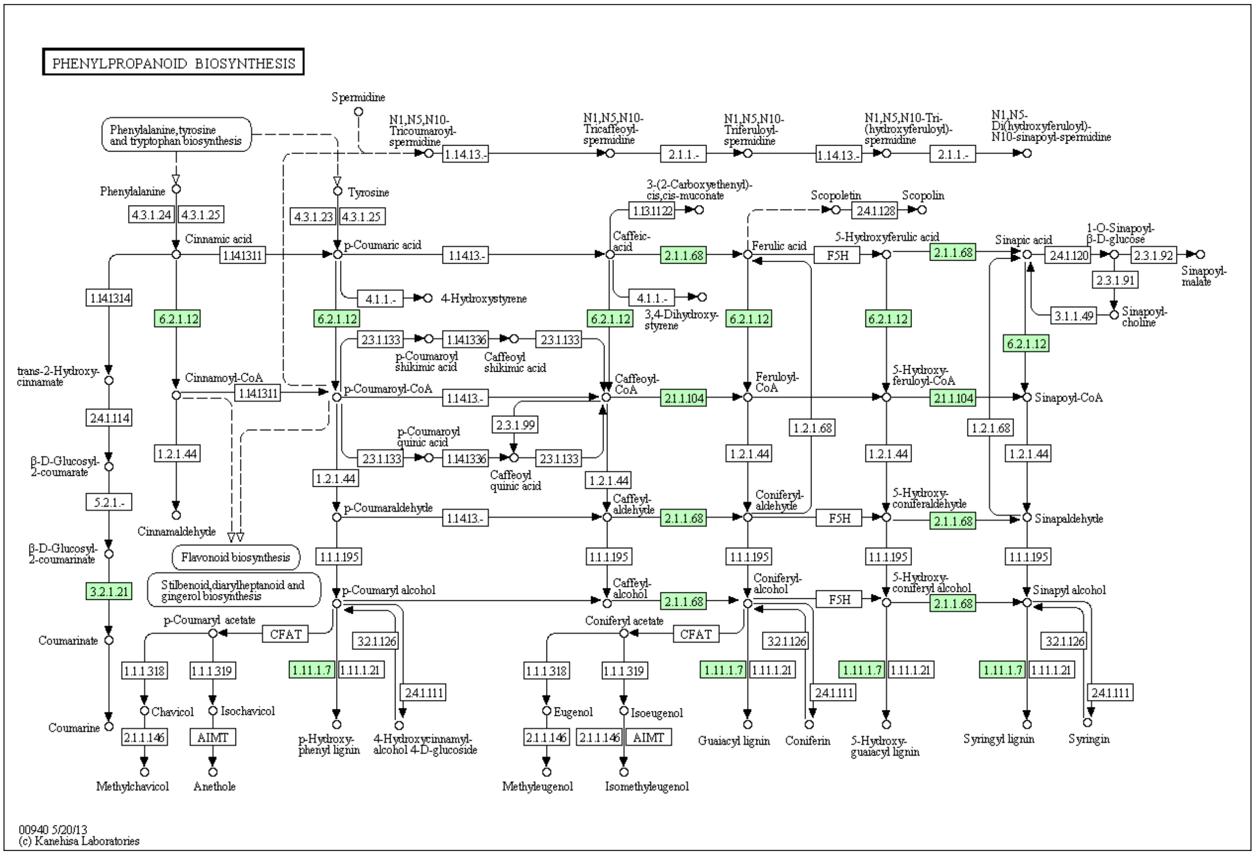

Supplemental Figure S5. Reconstruction of the flavonoid pathway using KEGG pathway [1-3] classification. Genes that were differentially expressed during the six time-points examined in *M. truncatula* macrosclereid cell development are shaded in green. This pathway

1. Kanehisa M, Furumichi M, Tanabe M, Sato Y, Morishima K. KEGG: new perspectives on genomes, pathways, diseases and drugs. Nucleic Acids Res. 2017;45(D1):D353-D61. doi: 10.1093/nar/gkw1092. PubMed PMID: 27899662; PubMed Central PMCID: PMCPMC5210567.
2. Kanehisa M, Goto S. KEGG: kyoto encyclopedia of genes and genomes. Nucleic Acids Res. 2000;28(1):27-30. PubMed PMID: 10592173; PubMed Central PMCID: PMCPMC102409.
3. Kanehisa M, Sato Y, Kawashima M, Furumichi M, Tanabe M. KEGG as a reference resource for gene and protein annotation. Nucleic Acids Res. 2016;44(D1):D457-62. doi: 10.1093/nar/gkv1070. PubMed PMID: 26476454; PubMed Central PMCID: PMCPMC4702792.
